# Modelling Vulnerability and Range Shifts in Ant Communities Responding to Future Global Warming in Temperate Forests

**DOI:** 10.1371/journal.pone.0159795

**Published:** 2016-08-09

**Authors:** Tae-Sung Kwon, Fengqing Li, Sung-Soo Kim, Jung Hwa Chun, Young-Seuk Park

**Affiliations:** 1 Forest Insect Pests and Diseases Division, National Institute of Forest Science, Dongdaemun, Seoul 02455, Republic of Korea; 2 Department of Life and Nanopharmaceutical Sciences and Department of Biology, Kyung Hee University, Dongdaemun, Seoul 02447, Republic of Korea; 3 Research Institute for East Asian Environment and Biology, Arisuro 25 Nagil 24, Gangdong, Seoul 05207, Republic of Korea; 4 Division of Forest Ecology, National Institute of Forest Science, Seoul 02455, Republic of Korea; University of Colorado, UNITED STATES

## Abstract

Global warming is likely leading to species’ distributional shifts, resulting in changes in local community compositions and diversity patterns. In this study, we applied species distribution models to evaluate the potential impacts of temperature increase on ant communities in Korean temperate forests, by testing hypotheses that 1) the risk of extinction of forest ant species would increase over time, and 2) the changes in species distribution ranges could drive upward movements of ant communities and further alter patterns of species richness. We sampled ant communities at 335 evenly distributed sites across South Korea and modelled the future distribution range for each species using generalized additive models. To account for spatial autocorrelation, autocovariate regressions were conducted prior to generalized additive models. Among 29 common ant species, 12 species were estimated to shrink their suitable geographic areas, whereas five species would benefit from future global warming. Species richness was highest at low altitudes in the current period, and it was projected to be highest at the mid-altitudes in the 2080s, resulting in an upward movement of 4.9 m yr^−1^. This altered the altitudinal pattern of species richness from a monotonic-decrease curve (common in temperate regions) to a bell-shaped curve (common in tropical regions). Overall, ant communities in temperate forests are vulnerable to the on-going global warming and their altitudinal movements are similar to other faunal communities.

## Introduction

Global warming has produced clear fingerprints on biosphere [1–4]. To cope with warming effects, the poleward and upward shifts in species’ distributional ranges have been increasingly observed across a wide range of taxonomic groups, e.g., plants, insects, birds, and benthic invertebrates [1, 5–9]. The range shifts caused by global warming would radically shape the compositions of biotic communities and local diversity, as well as the functions and services of local ecosystems [3]. For example, it is expected that global warming will decrease the fitness of tropical insects but increase that of temperate insects [10]. However, the changes in fitness in temperate regions vary greatly among species due to their differences in ecological, physiological, and evolutionary characteristics.

Ants are ideal organisms for testing or predicting the impacts of global warming on biosphere because they are dominant insects in most terrestrial ecosystems worldwide [11]. As keystone organisms, ant communities are closely related to other insects, plants, microorganisms, and soil, and the changes in ant community compositions are likely to cause great cascade impacts on terrestrial ecosystems [11]. Several studies have projected the effects of global warming on ant species, e.g., distribution of invasive ant species such as *Linepithema humile* [12], upward movement of *Aphaenogaster* species [13], global patterns of ant species richness [14], and ant foraging activities and community metrics [15]. However, up to date, no study has investigated the climate-induced risk of extinctions and upward movements of ant species incorporating the whole surveyed communities.

Although both precipitation and temperature are important climatic factors determining species distribution patterns, temperature has been used primarily to evaluate the effects of global warming on organisms [3, 7, 16] because precipitation varies in time and space and its prediction is more difficult compared with temperature. For example, in North America an upward shift of ants was not related with the change in precipitation but significantly related with the rise in temperature [13], and similarly the distribution of ants in South Korea was mainly determined by temperature because other climatic factors are relatively homogeneous in this region [17].

As temperature rises, ants may move upwards in mountain forests but the projected responses of ant species vary along the altitudinal gradient. In our previous study, we examined the response of individual ant species to temperature increase at the 2060s, where the abundance of 11 species was expected to decrease, whereas five species were expected to increase [17]. In this study, we developed species distribution models to further evaluate the potential impacts of temperature increase on the risk of extinctions and upward movements of ant species in the context of community and particularly aimed to test the following hypotheses: 1) the risk of extinction of forest ant species would increase over time, and 2) the changes in ant species distribution ranges could drive upward movements of ant species and further alter the patterns of species richness.

## Methods and Materials

### Ethical statement

No permits were required to access sampling sites and to conduct field work. However field sampling was supervised by Korea Forest Research Institute (Seoul, Republic of Korea). The field studies did not involve endangered or protected species.

### Field survey

**Fig 1:**
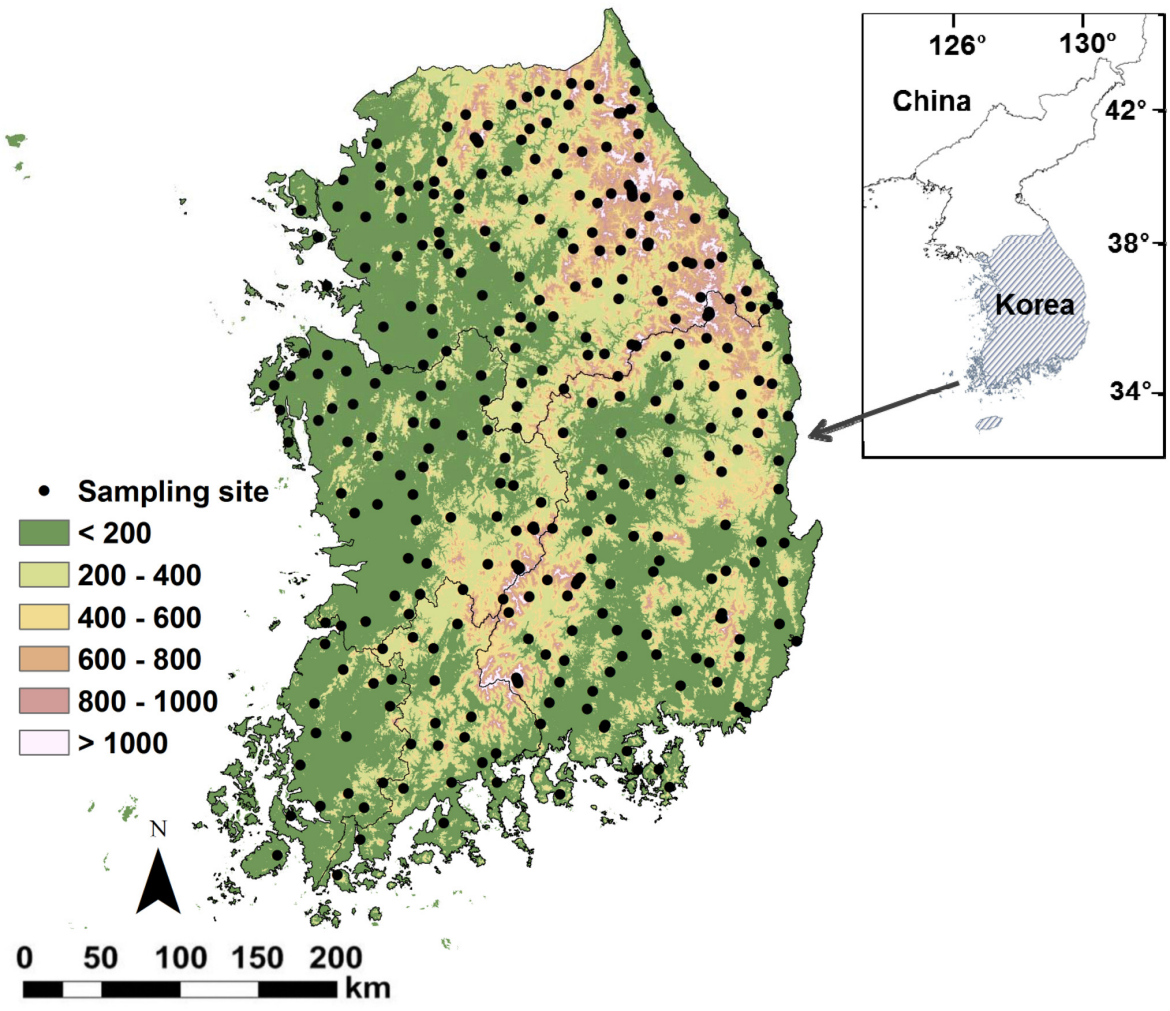
Distribution of the sampling sites and the digital elevation patterns.

Ants were surveyed at 335 sampling sites in the undisturbed forests or bush lands (mountain tops) with limited anthropogenic disturbance on a nationwide scale in South Korea (Fig 1). The sampling sites were composed of trees > 30 years with well-developed understory vegetation. Among the 335 sampling sites, 195 were located in deciduous forests, 129 were in coniferous forests, one was in a mixed forest, and the remaining ten were in bush lands (mountain tops).

Ants were collected with pitfall traps consisting of a plastic cup (depth: 6.3 cm; diameter: 9.5 cm) with polyethylene glycol as a preservative. The pitfall traps were placed along a transect line and each trap was 5 m apart. In total, ten pitfall traps were installed at each sampling site for ten days from mid-May to mid-September (i.e., active period for ant foraging in South Korea) over 2007–2009. Diversity and species composition of ground foraging ants are not different among sampling times [18]. All ant specimens were sorted from debris in the laboratory, preserved in 80% ethanol, and deposited in the Forest Ecology Laboratory of the National Institute of Forest Science of Korea.

The specimens were identified to species level based on Kwon *et al*. [19], except *Lasius japonicus* and *L*. *alienus*, which were the most common and abundant species in the genus *Lasius*. These two species were treated as a species group (*Lasius* spp. [*japonicus* + *alienus*]) because the intermediate forms of these two species were found frequently. Fifty-seven ant species were collected, and 29 of the most common species (>5% occurrence frequency) were selected to evaluate the potential impacts of temperature increase on ant communities (S1 Table).

### Temperature data

Temperature data at each sampling site was extracted from digital climate maps produced by the Korea Meteorological Administration (KMA) and National Center for Agrometeorology. The output of the global climate model (ECHO-G) was downscaled using the Mesoscale Model 5 to produce a resolution of 27 km regional climate change scenario (http://ccs.kma.go.kr). The results of regional climate model were further downscaled statistically to generate the Applied Climate Data in Korea (30 m resolution) for the agricultural studies [20–22]. In the regional downscaling procedure, interpolated climatic variables were obtained from a comprehensive mapping project, including 76 national permanent weather stations and 432 automatic weather systems [22, 23]. Korea Forest Research Institute verified these climatic models including mean annual precipitation, temperature, and solar radiation with commercial climatic devices [23–25]. Although several emission scenarios were considered in this climatic database, data with A1B emission scenario (720 ppm atmospheric CO_2_ in the 2100s) was selected in our study because it is defined as not relying too heavily on one particular energy source, but on the assumption that similar improvement rates apply to all energy supply and end-use technologies [16].

The average decadal air temperature was not available in the KMA database, we therefore selected the decadal (2000s–2080s) minimum and maximum air temperature data (resolution: 30 × 30 m) to calculate the average decadal air temperature using the Geographic Information System program (ArcGIS 9.3, ESRI). For a given sampling site, the average decadal air temperature was calculated as follows [16]: 1) the sampling site was located in a grid (30 × 30 m), and this grid together with its connected eight surrounding grids formed a nine-cell coenobium; 2) the average maximum and minimum decadal air temperatures in these nine grids were then calculated based on the arithmetic average; and 3) the average decadal air temperature was finally calculated as the arithmetic average of the maximum and minimum decadal air temperatures. The predicted average temperature increases were 0.5°C, 1.2°C, 2.4°C, and 3.5°C by the 2020s, 2040s, 2060s, and 2080s, relative to the baseline period of the 2000s, respectively.

### Risk rates for forest ants

Two steps were taken to evaluate the risk rates for forest ants in our study. First, a weighted averaging regression model (WARM) was used to quantify the optimal and tolerance temperatures. The optimal temperature for each species was calculated as the mean air temperature weighted by the abundance of this taxon at all sampling sites according to eq 1 [26]:

$$WA=\frac{\sum_{i=1}^{n}x_{i}\times y_{ij}}{\sum_{i=1}^{n}y_{ij}}$$ (1)

where *WA* is the weighted average (estimate of species optima), *x*_*i*_ is the air temperature at site *i*, and *y*_*ij*_ is the abundance of species *j* at site *i*.

The tolerance temperature was calculated as the weighted standard deviation of taxon abundance at all sampling sites according to eq 2 [26]:

$$TOL=\sqrt{\frac{\sum_{i=1}^{n}{\left(x_{i}-WA\right)}^{2}\times y_{ij}}{\sum_{i=1}^{n}y_{ij}}}$$ (2)

where *TOL* is the tolerance to air temperature for species *j*.

WARM was developed using C2 program ver. 1.6.7 [27]. The explanation power (*R*^*2*^) of air temperature to the community variations was used to evaluate the accuracy of WARM. Errors associated with model inferences were estimated by bootstrapping with 1000 cycles. The WARM model explained two-thirds regression variations using the bootstrapping approach (*R*^*2*^ = 0.66, average bias = 0.01, root-mean-square error = 1.52), the WARM results therefore could be used. To show the altitudinal range of the common ant species, the optimal and tolerance altitudes for each species were calculated following the same approach as air temperature.

Second, species’ maximum air temperature tolerances were calculated as the sum of optimal and tolerance values. If this value was smaller than the lowest air temperature evaluated with the A1B scenario in the study area, the species was defined as at risk due to the absence of the suitable thermal habitats. The percentage of risk rate for each future decade (2020s, 2040s, 2060s, and 2080s) was calculated as the proportion of number of risk state species to the total number of species recorded at the sampling sites. The lowest air temperature for each decade was arbitrarily defined as the first percentile of the temperature in the lateral buffer zones (i.e., 100 m adjacent to the sampling site). We analyzed the relationship between altitude and the risk of species extinction in the 2080s for four different groups 1) all species, 2) species with more than 1% of occurrence frequency, 3) species with more than 5% of occurrence frequency, and 4) species with more than 10% of occurrence frequency using logistic regressions. Additionally, the risk rates for forest ant communities in six altitude groups (≤200, 200–400, 400–600, 600–800, 800–1000, and > 1000 m) were quantified using the above methods. The number of sampling sites in each altitude group was 157, 77, 27, 25, 21, and 28, respectively.

### Generalized additive model and autocovariate regression

Differed from our previous study [17], where polynomial regression models were used to predict the changes in abundance of ant species, our present study employed one of the most widely used species distribution models (SDMs), the generalized additive model (GAM), to predict future suitable geographic areas for each species based on the current species distribution patterns and temperature conditions in the current and future decades. GAM quantifies the relationship between independent variables and species abundance (continuous variable) based on known locations, and the results are used to estimate species distributions by comparing different independent variable layers. Nine independent variables indicating annual, winter, and summer meteorological condition (annual maximum, annual average, annual minimum, January maximum, January average, January minimum, July maximum, July average, and July minimum air temperature) were selected as input abiotic variables for GAMs. Although high correlations were detected between each pair-wised abiotic variables, we still choose all nine variables in the model because 1) considerably low predictive power was observed when we selected any one of nine variables to calibrate GAM; and 2) the effects of temperature variables on ant species are complicated and inconsistent, and only one or several temperature variables may fail to explain a large variation of ant species. GAM additionally uses a non-parametric smooth function to improve the predictive efficiency, as shown in eq 3 [28]:

$$g(\mu )=\alpha +\overset{n}{\underset{i=1}{\sum }}f_{i}(x_{i})$$ (3)

where *g(μ)* is the response (i.e., predicted presence/absence of a given species), *f*_*i*_ is the smooth function, *x*_*i*_ is the explanatory variable (i.e., temperature), *n* is the number of explanatory variables, and *α* is residual error.

A cross-validation approach was used to assess the predictive accuracy of individual GAM. First, the entire dataset was partitioned into a training set (70%) and testing set (30%). Second, models were calibrated using the training set and validated using the testing set. The goodness of fit of GAM was estimated based on the following indicators: a) area under the curve (AUC) statistics from threshold-independent receiver operating characteristic plots; and b) sensitivity and specificity, indicating the true positive rate (i.e., presence) and true negative rate (i.e., absence) are correctly projected [29, 30]. AUC ranges from 0.5 to 1, where 0.5 represents no discrimination and 1 represents perfect discrimination. Model predictive accuracy is acceptable when AUC > = 0.7 [31]. Sensitivity and specificity range from 0 to 1, where 0 represents bad prediction and 1 represents perfect prediction. The GAMs were performed using the statistical package “*gam*” [32] in R (http://cran.r-project.org).

Prior to GAMs, spatial autocorrelation test was conducted. The results showed a clear spatial autocorrelation among sampling sites using abundance data (Moran's I test, *P* < 0.0001). To account for the effect of spatial autocorrelation, autocovariate regression (AR) was employed [33, 34]. The generated eigenvectors from AR were then incorporated as additional predictors in GAM. The corrected spatial model using both AR and GAM was then used to predict the future distributions of individual ant species.

## Results

**Fig 2:**
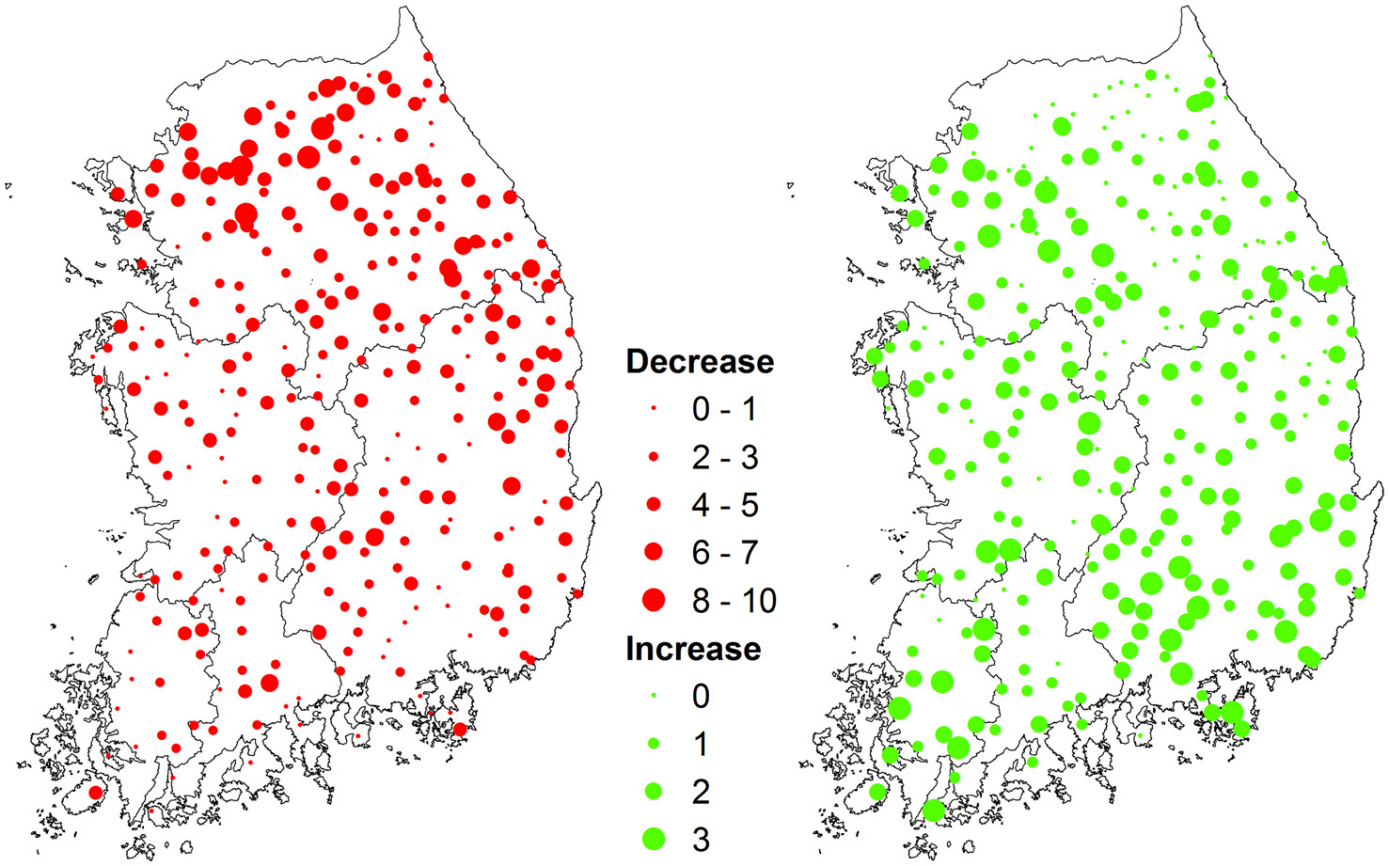
Maps of number of ant species expected to decrease (red) and increase (green) by the 2080s in Korea.

Relationships between altitude and risk of extinction of common ant species (> 5% occurrence frequency) were significantly positive (S1 Fig). Similar patterns were observed with those of all species and species with > 1% occurrence frequency, whereas no clear trends were observed with that of species with > 10% occurrence frequency (S1 Fig). The optimal altitude and air temperature for the selected species were 114–1080 m and 7.0–12.9°C, respectively (S2 Table). The indicators of predictive accuracy of GAMs ranged between 0.70 and 0.99 (average = 0.85), between 0.52 and 0.97 (average = 0.62), and between 0.59 and 1.00 (average = 0.84) for AUC, sensitivity, and specificity, respectively (S3 Table), indicating that the calibrated GAMs were reliable with relatively high goodness of fit. Among the 29 common species, 12 species were expected to decrease in the suitable geographic areas (decreased species), whereas five species were predicted to increase (increased species) (S2 Table). The remaining 12 species were predicted to have no significant changes in their suitable geographic areas (stable species). Overall, the group of decreased species was generally from high altitude ranges, e.g., north region (Fig 2).

**Fig 3:**
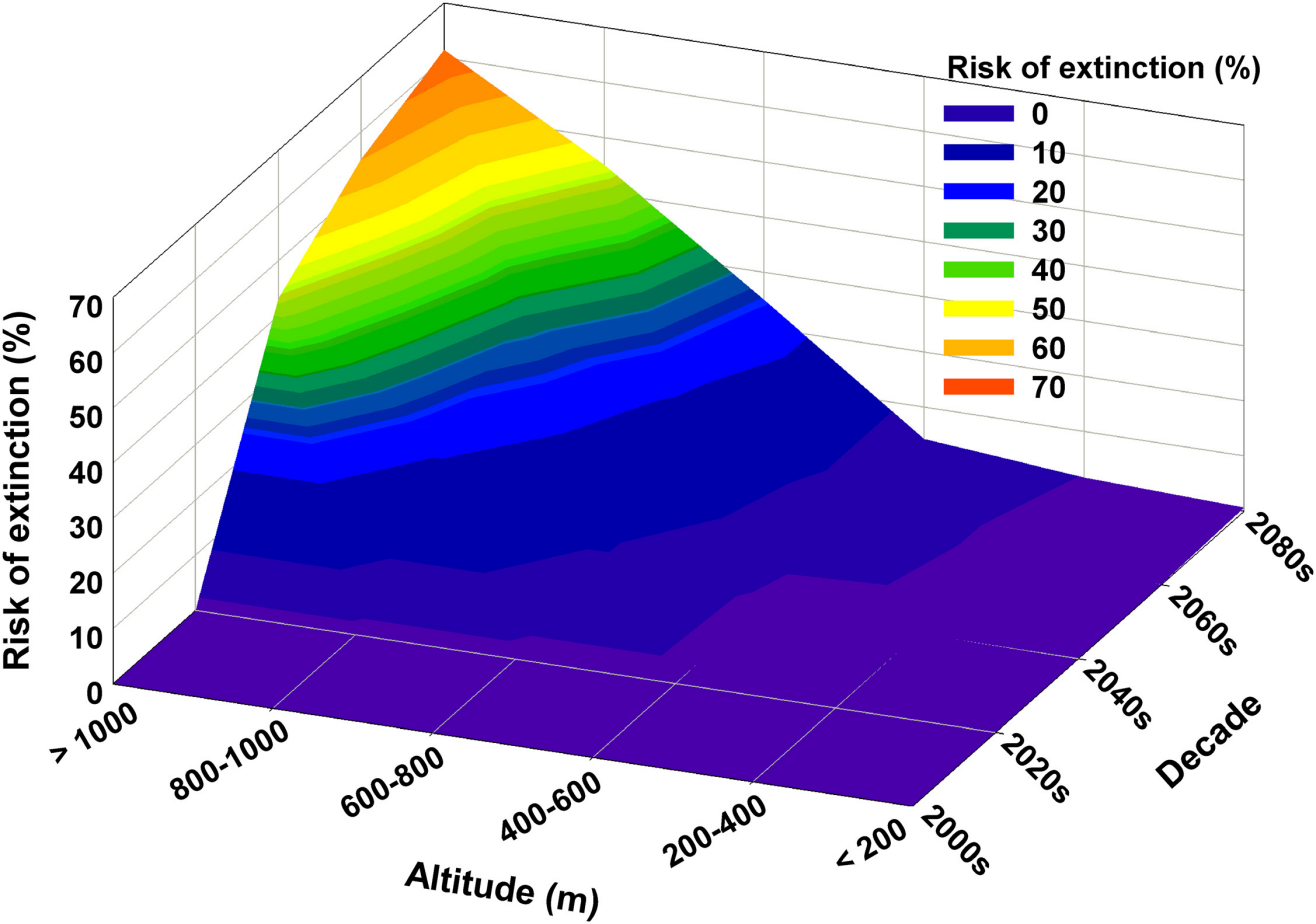
Temporal patterns of common ant species at risk of extinction across the altitude gradient.

A loss of biodiversity was projected in the 2040s and increased gradually until the 2080s, suggesting that the high altitude areas may have higher biodiversity loss than that in low altitude areas (Fig 3). The highland areas (i.e., > 1000 m) were expected to face the highest biodiversity loss by the 2080s of approximately 61.5%, whereas biodiversity in lowland areas (<400 m) was not seriously affected. Overall biodiversity loss across all altitudinal gradients by the 2080s was approximately 7.6%.

**Fig 4:**
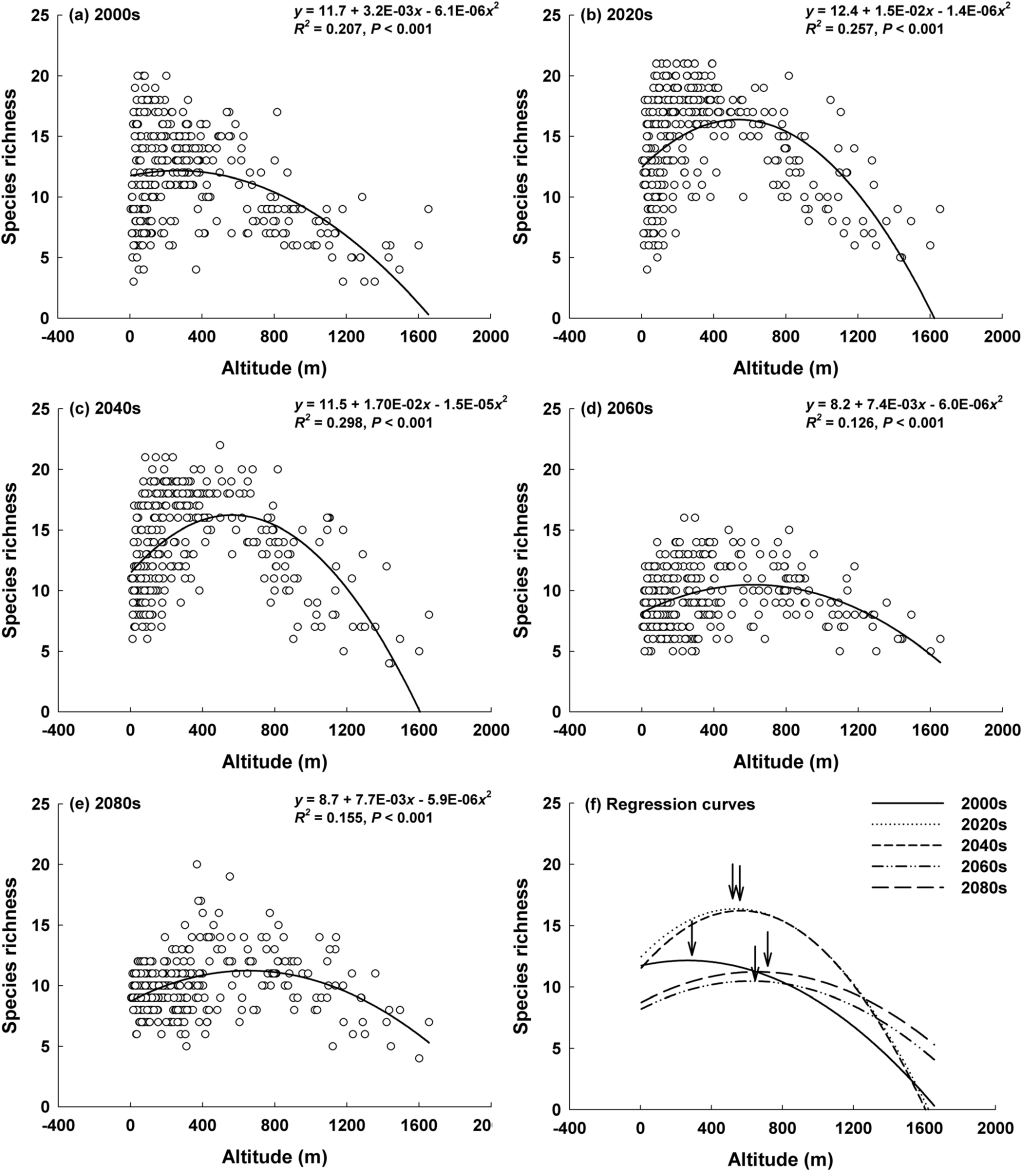
Projected richness of ant species in the (a) 2000s, (b) 2020s, (c) 2040s, (d) 2060s, (e) 2080s, and (f) the patterns of predicted curves. Arrow indicates the highest predicted species richness in each decade. Arrow sequence from left to right: 2000s, 2020s, 2040s, 2060s, and 2080s.

The location of the highest species richness was expected to continuously upward move from 263 m (the 2000s) to 656 m (the 2080s), representing an upward movement of 4.9 m yr^−1^ (Fig 4). The regression curves between the predicted species richness and altitude changed from a monotonic decrease response curve in the 2000s to a bell-shaped response curve in the future decades (Fig 4).

## Discussion

In this study, we estimated the future risk of extinction and upward movement of Korean ant species in response to the A1B emission scenario. Our projection suggests that ant species in temperate forests are vulnerable to global warming, and more ant species are expected to move to higher elevations. Due to these upward movements, ant species diversity is expected to increase in richness at high altitudes and decrease at low altitudes, and we determined that the altitudinal pattern of ant richness will likely switch gradually from a low altitude to a mid-altitude peak, and was prevalent in tropical regions. As keystone species, the upward movement of ant communities may lead to a mismatch of interactions (e.g., between phenology and food-web) among ants and plants as well as other insects.

### Risk of extinction

The projected responses of ant species to global warming may continue in the Korean Peninsula and the continuous increase in the risk of extinction observed covered all altitudinal gradients (confirming the first hypothesis). Cold-adapted ants are expected to eventually become extinct in South Korea because these currently mountain-dwelling species do not have alternative habitat to move up [35]. The abrupt decline in *Myrmica* species due to global warming in highland regions may severely affect competitive ant species (e.g., *Aphaenogaster japonica*), ground beetles, aphids, butterflies, and soil arthropods, leading to cascade effects on plants, birds, rats, snails, fungi, and mushrooms as well as other organisms in the Korean highlands [36, 37]. Overall, our projection is regionally applicable to ant fauna in East Asia, including North Korea, mid to northern Japan, and eastern to northeastern China, since these neighboring regions have ant faunas similar to those found in South Korea [38, 39].

Compared with ambient acclimated ants, forest ants generally have a higher physiological temperature tolerance, suggesting they might not be affected by warming. Particularly in temperate regions, insects are expected to be at low risk to global warming [40], with their predicted diversity predicted to increase in response to future global warming trends [10]. However, our observations indicated that there was a high risk of extinction for ant species. Specifically, we predicted that one-third of ant species will decrease in abundance in their distributional range even with leading to local extinction events such as those observed in dominant species in found in highland regions, such as *Myrmica kurokii* and *Stenamma owstoni* [17]. The projection in the abundance of 85 species of Korean spiders demonstrated that 80% (68 of 85 species) may decrease in abundance and distribution in the future [41]. Projections determined for aquatic insects also show similar patterns [16]. The cold-adapted species in the Korean Peninsula might have migrated from the northern region, whereas the warm-adapted species could have originated farther south [42], leading to in the peninsula effect on species diversity, characterized by an increase in richness along a latitudinal gradient. Due to this peninsular topographical effect, numerous cold-adapted species tend to decrease in abundance since they have lower thermal requirements compared to warm-adapted species [16]. However, it may be difficult for recently warm-adapted species to migrate and occupy vacant habitats on the Korean Peninsula due to geographical barriers, such as the sea. This may in turn cause insect fauna on the peninsula to become more vulnerable to global warming compared to the species that inhabit non-peninsular landmasses. For example, the risk of aquatic insect species extinction in two major catchments in the Korean Peninsula was expected to be 50.4%–54.6% by the end of this century [7].

### Shifts in species distribution

Ant community composition in the temperate forests in Korea were predicted to change rapidly. However, the expected responses among ant species were different according to the thermal origins of the species. The warm-adapted species acclimated to high optimal temperatures may increase in abundance in suitable geographic areas; whereas the cold-adapted species acclimated to low optimal temperatures may decrease in abundance. In the present study, nearly one-third of the Korean ant species surveyed are expected to decrease in abundance in suitable geographic areas in response to global warming, whereas one-fifth is expected to increase. This is in line with our previous study [17], in which we found that more species were expected to decrease in number rather than increase.

The distribution of the 29 ant species is predicted to shift upwards by 4.9 m yr^−1^ from the 2000s to the 2080s (confirming the second hypothesis), which was similar to what was found in butterflies in Europe [8]. The range shift in suitable geographic areas revealed that the highest species richness was projected to be at the low-altitudes in the 2000s and at the mid-altitudes in the 2080s. Previous studies have revealed that species richness peaks at mid-altitudes (where it is bell-shaped) in the tropics in a worldwide survey of leaf litter ant diversity, whereas it decreases continuously with altitude (monotonic decrease) in temperate regions [43, 44]. For example, Fisher [45] reported that ant richness peaks at a mid-elevations of approximately 800 m in four mountains in tropical Madagascar, whereas Sanders *et al*. [46] found that ant species richness is negatively related to temperature in deciduous forests along an altitudinal gradient in the temperate regions of the Great Smoky Mountains. Similarly, ant richness monotonically decreased along an altitude gradient in 12 high mountains in South Korea with the highest richness at approximately 160 m [44].

Interestingly, the cold-adapted ant species displayed the highest richness at mid-elevations [44]. Species richness of cold-water adapted organisms such as stoneflies and caddisflies were predicted to decrease monotonically as a function of altitude in Korean streams, whereas that of warm-water adapted organisms, such as dragonflies, was projected to increase [16]. Altitudinal diversity patterns may be determined by the thermal origins of the relevant species with the following patterns: the dominance of warm-adapted species leads to a monotonic-decrease, a balance between warm-adapted species and cold-adapted species leads to a bell-shaped pattern, and finally the dominance of cold-adapted species leads to a monotonic-increase [44]. These results indicate that an increase in warm-adapted species and a decrease of cold-adapted species may lead to a clear monotonic-decreasing pattern in the future. However, our projection revealed an altered distribution in species richness from a monotonic-decrease (in the 2000s) to a bell-shaped pattern (2060s–2080s) due to global warming, confirming the second hypothesis. This change might be caused by an upward shift in lowland (warm-adapted) species and a decrease in highland (cold-adapted) species across mid to high altitudes. To a certain extent, ant diversity in South Korea therefore is likely to change from a temperate pattern (characterized by a low-elevation peak) to a tropical pattern (with a mid-elevation peak) during the 2060s.

### Limitations of the study

Similar to most studies implementing SDMs, our study has some limitations springing from various sources, including the uncertainty of the previously determined models [47, 48]. First, SDM is a statistical-correlative forecast approach. An assumption of SDM is that species can move to all potentially suitable areas without limitations; however, in reality, a species may not occur at a suitable location because of the potential threats or challenges (e.g., dispersal barriers and habitat fragmentation) [49]. Concerning the uncertainty in the forecasting of species distribution, Buisson *et al*. [47] revealed that SDMs contribute to the highest variation expected in the projections. Through utilizing SDMs, Hof *et al*. [50] reported that the ratio of observed to predicted range size of European odonates ranged between 0.6 and 0.9, inferring that the uncertainty (i.e., 1 –predictive accuracy) of SDMs ranged from approximately 10% to 40%. In our case, our model uncertainty ranged between 0.15 (the mean of AUC) and 0.38 (the mean of sensitivity). We, therefore, deduced that the overestimation of ant species potential distribution areas is approximately 10%–40%.

Second, the simulated climate data also have uncertainty due to climate models. The uncertainty of the climate models originates from the input values of greenhouse gasses and also depends on how these models are weighted [48]. Although the climate models used in this study have been verified in several areas in Korea [23–25], uncertainty still exists, which is likely to influence the output of the SDMs implemented.

Third, the modeled risk of extinction for ant species may also be overestimated due to species’ ecological and evolutionary adaptations to environmental changes [49]. For example, forest ant species have much higher physiological temperature tolerance than other fauna, which may mitigate the effects of global warming on ant species.

Fourth, climate change may impact the physiology of organisms as well as their habitats. In this study, we focused only on physiological aspects affecting species distribution, while due to a lack of data, other potentially important aspects, such as local habitats (e.g., vegetation and soil types) and interactions were not accounted for in our species distribution model [49]. When we consider a change of habitat and other possible factors, the results of the model might be influenced. For example, it has been reported that recent forest canopy closure in northern-hemisphere temperate forests has buffered the impact of global warming on plant communities, which delays changes in community composition [51]. In future studies, it is, therefore, necessary to simultaneously assess the impacts of projected climate change on the habitats and, in turn, the changes in habitat influencing species distribution. Despite these potential limitations, our results are comparable with other studies, and provide a first glance at the possible response of ant species to future global warming.

In response to global warming, species can shift their distribution ranges to move to favorable habitats [1, 16, 52], or might persist in their original habitats invoking genetic strategies such as phenotypic plasticity or rapid evolutionary adaptation [53, 54]. Ant species can quickly adapt to environmental change, and in particular, they can avoid extremely low or high temperatures through physiological and/or behavioral adaption. For example, most ground foraging ant species overwinter in deep soils. In addition to temperature control, length of foraging time might be another important factor constraining species distribution. However, we did not consider the ability of species to adapt to global warming in the present study, and our results could be altered if the ability of each species to adapt is implemented in the model. Therefore, follow-up studies on the response of the entire community to global warming are required and should incorporate the ability of individual species to adapt to their environment.

## Supporting Information

S1 Fig Logistic relationships between altitude and the proportion of taxa at risk of extinction in the 2080s for (a) all taxa, (b) > 1%, (c) > 5%, and (d) > 10% frequent occurrence. (PDF)

S1 Table Occurrence of 29 common ant species used in the study. (PDF)

S2 Table Summary of altitude and temperature conditions and statistics of linear regressions between the proportion of predicted suitable geographic areas and period. (PDF)

S3 Table Summary of the calibrated generalized additive models (GAM; corrected by spatial autocorrelation) and the indicators of model accuracy. (PDF)
