# Supplementary material for: Modelling Vulnerability and Range Shifts in Ant Communities Responding to Future Global Warming in Temperate Forests
Source: PLoS One. 2016 Aug 9;11(8):e0159795. doi: 10.1371/journal.pone.0159795 (PMC4978472; doi:10.1371/journal.pone.0159795)

S1 Fig. Logistic relationships between altitude and the proportion of taxa at risk of extinction in the 2080s for (a) all taxa, (b) > 1%, (c) > 5%, and (d) > 10% frequent occurrence. The risk of extinction is defined as the proportion of species extinct in each site.

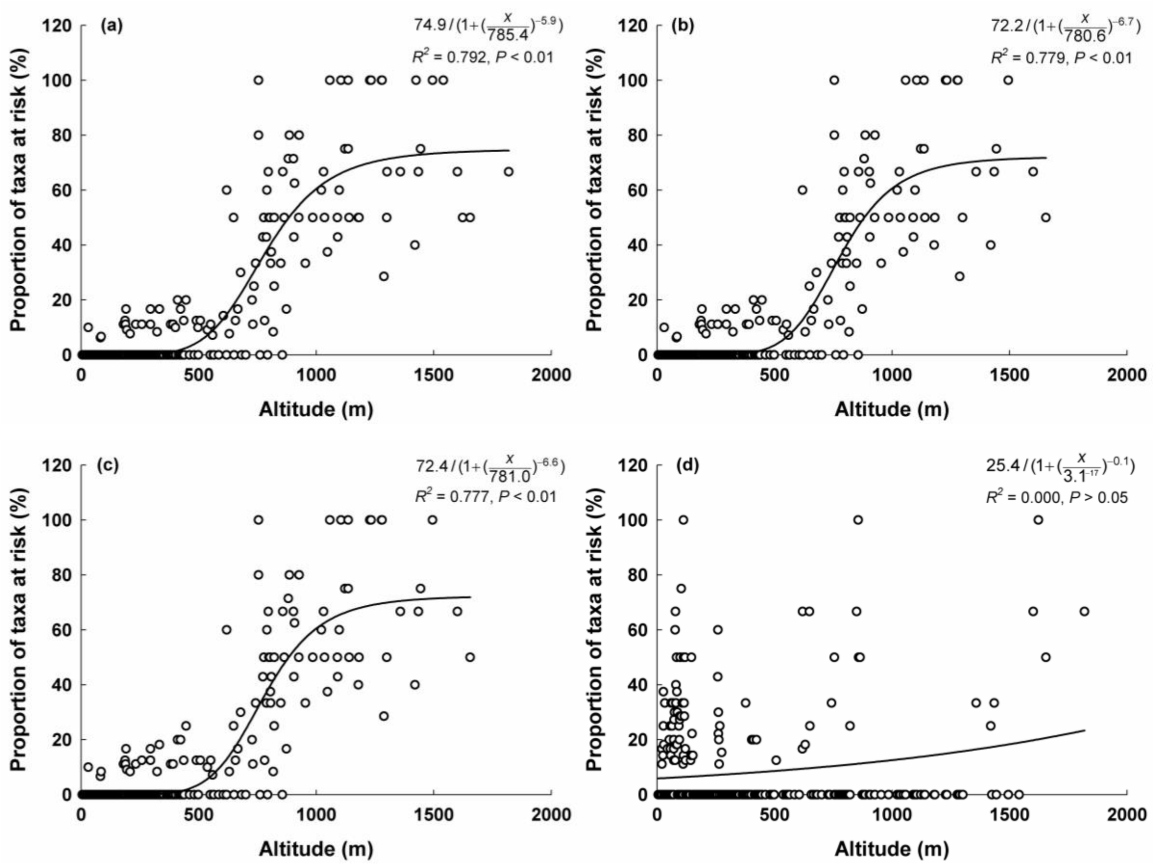

Supplement: S1 Fig — (PDF) [file pone.0159795.s001.pdf]
