# Supplementary material for: Modelling Vulnerability and Range Shifts in Ant Communities Responding to Future Global Warming in Temperate Forests
Source: PLoS One. 2016 Aug 9;11(8):e0159795. doi: 10.1371/journal.pone.0159795 (PMC4978472; doi:10.1371/journal.pone.0159795)
Supplement: S1 Table — (PDF) [file pone.0159795.s002.pdf]

S1 Table. Occurrence of 29 common ant species used in the study.

Latitude	Longitude	Site_name	sp1	sp2	sp3	sp4	sp5	sp6	sp7	sp8	sp9	sp10	sp11	sp12	sp13	sp14	sp15	sp16	sp17	sp18	sp19	sp20	sp21	sp22	sp23	sp24	sp25	sp26	sp27	sp28	sp29
458744	149284	R001	1	1	0	0	1	1	1	1	0	1	0	0	0	0	1	1	0	0	0	1	1	0	0	0	0	0	0	0	0
458818	149211	R002	0	1	0	0	0	0	0	1	0	1	0	0	0	1	1	1	0	0	0	0	1	0	0	0	0	0	0	0	0
460828	169213	R003	0	0	0	0	0	1	1	1	0	0	1	0	0	1	1	0	0	1	1	0	1	0	0	0	0	0	0	0	0
475492	172155	R004	1	1	0	0	1	1	1	1	0	0	0	0	0	1	1	1	0	0	0	1	1	0	0	0	0	0	0	0	1
482184	192548	R005	0	0	0	0	1	0	0	1	0	0	0	0	1	1	1	1	0	1	0	0	1	1	0	0	0	1	0	1	0
495138	190395	R006	0	1	0	1	1	1	1	1	0	1	1	0	0	0	1	1	0	0	1	0	1	0	0	0	0	0	0	0	0
504323	228693	R007	0	0	1	0	1	1	0	1	1	0	0	0	1	0	1	1	0	0	0	1	1	0	0	0	0	1	0	0	0
444044	158400	R008	0	1	0	0	1	0	1	1	0	1	1	1	0	0	1	1	0	1	1	1	1	1	0	0	0	0	0	0	0
455271	184461	R009	0	0	0	0	1	0	1	1	0	1	1	0	0	1	1	0	0	1	0	1	1	0	0	0	0	0	0	0	0
472378	192400	R010	0	1	1	1	1	1	1	1	0	1	0	0	0	1	1	1	0	0	1	1	1	1	0	0	0	0	0	0	1
469447	202936	R011	0	1	1	0	0	0	1	1	1	0	0	0	0	1	1	1	0	0	1	1	1	0	0	0	0	0	0	1	0
472191	213034	R012	0	0	0	0	1	1	0	1	0	0	0	0	0	0	1	1	0	0	1	1	1	0	0	0	0	0	0	1	0
485536	226066	R013	0	1	0	0	1	1	0	1	0	0	1	0	1	0	1	1	0	0	0	0	0	0	0	0	0	1	1	1	0
495658	245744	R014	0	1	0	0	1	1	0	1	0	0	0	0	0	0	1	0	0	0	0	1	0	0	0	0	0	0	0	0	0
496786	245135	R015	0	0	0	0	1	0	0	1	0	0	0	0	0	0	0	0	0	0	0	1	0	0	0	0	1	0	0	0	0
497538	244664	R016	0	1	0	1	0	0	0	1	0	0	0	0	0	0	0	0	0	0	0	1	0	0	0	0	1	0	0	0	0
497553	244571	R017	0	0	0	0	0	0	0	0	0	0	0	0	0	0	0	0	1	0	0	1	0	0	0	1	1	0	1	0	0
497553	244572	R018	0	0	0	0	0	0	0	0	0	0	0	0	0	0	0	0	1	0	0	0	0	0	0	1	1	0	0	0	0
498607	243824	R019	0	0	0	0	0	1	0	0	0	0	0	0	0	0	0	1	0	0	0	0	0	0	0	1	1	0	0	0	0
427540	184282	R020	0	0	0	0	0	0	1	1	0	0	0	0	1	1	1	0	0	0	0	0	1	0	0	0	0	0	0	0	0
433798	201529	R021	0	1	1	0	0	0	1	1	0	1	0	0	0	0	1	1	0	0	0	0	1	1	0	0	0	1	0	0	1
454493	203817	R022	0	0	0	0	1	0	1	1	0	0	0	0	0	0	1	0	0	0	0	1	1	0	0	0	0	0	0	0	1
467695	221381	R023	0	0	0	0	1	1	1	1	0	1	0	0	1	0	1	0	0	1	1	0	1	0	0	0	0	0	0	0	0
474564	221657	R024	0	0	1	0	1	1	0	1	0	0	0	1	0	0	0	1	0	0	1	1	1	1	0	0	0	0	1	1	0
467536	235219	R025	0	1	0	0	0	0	0	1	0	0	1	0	0	0	1	1	0	1	0	1	1	0	0	0	0	0	0	0	0
417624	163354	R026	1	0	0	0	0	0	0	1	0	1	0	0	0	1	1	1	0	1	0	1	1	0	0	0	0	0	0	0	0
439786	215172	R027	0	1	1	0	0	1	0	1	0	0	0	0	0	0	1	0	0	0	0	1	1	1	0	0	0	0	0	1	0
446959	224319	R028	0	1	0	0	1	1	0	1	0	1	1	0	0	1	1	1	0	0	1	1	1	1	0	0	0	1	0	1	0
440219	224789	R029	0	1	0	0	1	0	1	1	0	1	0	0	0	0	1	1	0	0	1	0	0	0	0	0	0	0	0	1	0
447573	249377	R030	0	1	1	0	1	0	0	1	0	1	0	0	0	0	1	1	0	0	0	1	1	1	1	0	0	0	0	0	0
435344	229140	R031	0	1	0	0	0	0	0	0	0	0	0	0	1	0	1	0	0	0	1	1	1	1	0	0	0	0	0	1	0
439015	254739	R032	0	0	0	0	1	0	1	1	0	1	0	0	1	1	1	0	0	0	0	0	1	0	0	0	0	0	0	0	0
424916	236373	R033	0	0	0	0	0	0	1	1	0	1	0	0	0	0	1	1	0	0	1	0	1	0	0	0	0	0	0	0	1
405041	220481	R034	0	0	0	0	0	0	0	1	0	0	0	0	0	1	1	0	0	0	0	1	1	1	0	0	0	1	0	0	0
391883	220822	R035	0	0	0	0	0	0	1	1	0	0	0	0	0	1	1	1	0	0	0	0	1	0	0	0	0	0	0	0	0
382186	228325	R036	1	1	1	0	0	0	1	1	0	0	0	0	0	1	1	1	1	0	0	1	1	0	1	0	0	0	0	0	0
511081	238953	R037	0	0	0	1	0	0	0	1	0	0	0	0	1	0	0	0	0	0	0	0	0	0	0	0	0	0	1	0	0
505254	250756	R038	0	1	1	0	0	0	0	1	0	0	0	0	0	1	1	0	0	0	0	0	1	1	0	0	0	0	0	0	0
516360	263601	R039	0	1	0	0	1	1	0	1	0	0	0	0	0	1	1	0	0	0	0	0	1	0	0	0	0	0	0	0	0

520713	271978	R040	0	0	0	0	1	1	0	1	1	0	0	0	0	0	1	1	0	0	0	0	1	0	0	0	0	0	1	0	0
523635	278864	R041	0	0	1	0	1	1	0	1	0	0	0	0	1	1	1	0	0	0	0	1	1	0	0	0	0	1	0	0	0
497181	269086	R042	0	1	1	0	1	1	0	1	1	0	0	0	1	1	1	1	0	0	1	1	1	1	1	0	0	0	0	0	0
503269	273544	R043	0	1	0	0	0	1	0	1	0	0	0	0	0	0	1	0	0	0	0	0	1	0	0	0	0	0	0	0	0
506555	282791	R044	0	1	0	0	1	1	0	1	0	0	0	0	0	1	1	1	0	0	1	1	1	0	0	0	0	0	0	0	0
521921	287928	R045	0	0	0	0	1	0	0	1	0	0	0	0	0	0	1	0	0	0	0	0	1	0	0	0	0	0	1	0	0
516230	294869	R046	0	1	0	1	1	1	0	1	0	0	0	0	0	1	1	1	0	0	0	1	1	1	0	0	0	0	1	0	0
490752	302096	R047	0	0	0	0	0	0	0	1	0	0	0	0	0	0	1	0	0	0	0	0	0	0	0	0	0	0	0	0	0
528018	296404	R048	0	0	0	0	0	0	0	0	0	0	0	0	0	0	0	0	0	0	0	1	0	0	0	1	0	0	0	0	0
526999	305928	R049	0	1	0	1	1	0	0	1	1	0	0	0	1	0	1	0	0	0	0	1	1	0	0	0	0	0	0	0	0
539101	331019	R050	0	0	0	0	1	1	0	1	0	0	0	0	0	0	1	0	0	0	0	0	1	0	1	0	0	0	0	0	0
478711	247384	R051	0	1	0	0	1	0	0	1	0	1	0	0	0	1	1	1	0	0	0	1	1	1	0	0	0	0	0	0	0
480278	261080	R052	0	1	1	0	1	0	1	1	1	1	1	0	1	1	1	1	0	0	1	1	1	1	0	0	0	0	0	0	0
486647	276561	R053	0	1	1	0	0	1	0	1	0	1	0	0	0	0	1	0	0	0	0	1	0	0	0	0	0	0	0	0	0
478547	288503	R054	0	1	0	0	0	0	1	1	0	0	0	0	0	0	1	0	0	0	0	0	1	0	0	0	0	0	1	0	0
492664	291958	R055	0	1	0	0	0	0	0	1	0	0	0	0	0	1	1	0	0	0	0	0	1	0	0	0	0	0	0	0	0
519554	311165	R056	0	1	0	0	1	0	0	1	0	0	0	0	0	0	1	1	0	0	0	1	1	0	0	0	0	0	1	0	0
523763	330771	R057	0	1	0	1	0	0	0	1	0	1	0	0	0	0	1	0	0	0	1	0	1	1	0	0	0	0	0	0	0
459738	234941	R058	0	0	0	0	0	0	1	1	0	1	0	0	1	1	1	1	0	0	0	1	1	1	0	0	0	0	0	0	1
464763	269957	R059	0	0	0	0	0	0	0	1	0	0	0	0	0	1	1	1	0	0	0	1	1	1	0	0	0	0	1	0	0
453948	279215	R060	0	1	1	0	1	1	1	1	0	0	0	0	0	0	1	1	0	0	0	1	1	0	0	0	0	0	0	0	0
466981	300772	R061	1	0	0	0	1	0	1	1	1	0	0	0	0	1	1	1	0	0	0	0	1	1	1	0	0	1	0	0	0
462710	310411	R062	0	1	0	0	0	0	0	1	0	0	0	0	1	0	1	1	0	0	0	1	0	0	0	0	0	1	1	0	0
493390	315425	R063	0	1	0	0	1	0	1	1	1	0	0	0	0	0	1	0	0	0	0	1	1	0	0	0	0	0	1	0	0
487516	333179	R064	0	0	0	0	1	0	0	0	0	0	0	0	0	0	0	0	1	0	0	0	0	0	0	1	0	0	0	0	0
500448	332674	R065	0	0	0	0	0	0	0	1	0	0	0	0	0	0	1	0	0	0	0	0	0	0	0	0	0	0	0	0	0
511576	322069	R066	0	0	0	0	0	0	0	0	0	0	0	0	0	0	0	0	1	0	0	1	0	0	0	1	1	0	1	0	0
511659	323513	R067	0	0	0	0	0	1	0	0	1	0	0	0	0	0	0	0	0	0	0	0	0	0	0	1	1	0	1	1	0
511998	323708	R068	0	0	0	0	0	0	0	0	0	0	0	0	0	0	0	0	0	0	0	0	0	0	0	1	1	0	1	0	0
513907	328462	R069	0	0	0	0	0	1	0	0	0	0	0	0	0	0	0	0	0	0	0	0	0	0	0	1	1	0	0	0	0
467796	317864	R070	0	0	0	0	1	0	0	1	0	0	0	0	0	0	1	0	0	0	0	1	0	0	0	1	0	0	1	1	0
466380	329529	R071	0	0	0	0	1	0	0	0	0	0	0	0	0	0	1	0	0	0	0	1	0	0	0	1	0	0	1	1	0
466975	329375	R072	0	0	0	0	1	0	0	1	0	0	0	0	0	0	1	0	1	0	0	1	0	0	0	1	0	0	1	1	0
467790	329397	R073	0	0	0	0	1	0	0	1	0	0	0	0	1	0	0	0	1	0	0	1	0	0	0	1	1	0	0	1	0
469075	329367	R074	0	0	0	0	0	0	0	0	0	0	0	0	0	0	0	0	1	0	0	0	0	0	0	1	1	0	1	0	0
470546	329138	R075	0	0	0	0	0	1	0	0	0	0	0	0	0	0	0	0	1	0	0	0	0	0	0	1	1	0	0	0	0
472575	327583	R076	0	0	0	0	1	0	0	0	0	0	0	0	0	0	0	0	1	0	0	1	0	0	0	1	1	0	1	1	0
465702	336314	R077	0	1	0	1	1	0	0	1	0	0	0	0	0	0	1	0	0	0	0	1	0	0	0	0	0	0	0	0	0
422617	268101	R078	1	1	0	0	1	0	1	1	0	1	0	0	0	1	1	1	0	0	1	1	1	1	0	0	0	0	0	0	1
412655	247860	R079	0	0	0	0	1	0	1	0	0	0	0	0	0	0	1	1	0	0	0	0	1	0	0	0	0	0	0	0	0
410068	278895	R080	0	0	0	0	1	0	1	1	1	0	0	0	0	1	1	0	0	0	0	0	1	0	0	0	0	0	0	0	0

417214	298386	R081	0	1	0	1	0	0	0	1	0	0	0	0	0	0	1	1	0	1	0	1	1	0	1	0	0	0	0	0	0
436805	311722	R082	0	1	0	0	0	0	0	1	0	0	0	0	0	0	1	0	0	0	0	1	0	0	0	1	0	0	1	0	0
419378	310262	R083	0	0	0	0	0	0	0	1	0	0	0	0	1	0	1	0	0	0	0	1	1	0	0	0	0	0	0	0	0
436964	323177	R084	0	1	0	1	0	0	1	1	1	0	0	0	0	0	1	0	0	0	0	0	0	0	0	0	0	0	0	0	0
421438	324014	R085	0	0	0	0	1	0	0	0	1	0	0	0	0	0	1	0	0	0	1	0	1	0	0	0	0	0	0	0	0
439334	337921	R086	0	0	0	0	0	0	0	1	0	0	0	0	0	0	0	0	1	0	0	1	0	0	0	1	1	0	0	1	0
439590	337892	R087	0	0	0	0	0	0	0	0	0	0	0	0	0	0	0	0	1	0	0	1	0	0	0	1	1	0	0	1	0
440019	337890	R088	0	0	0	0	0	0	0	0	0	0	0	0	0	0	1	0	0	0	0	1	0	0	0	1	1	0	1	0	0
440657	337690	R089	0	0	0	0	0	0	0	0	0	0	0	0	0	0	0	0	1	0	0	0	0	0	0	1	1	0	0	0	0
441045	338249	R090	0	1	0	0	0	0	0	0	0	0	0	0	0	0	0	0	0	0	0	0	0	0	0	1	1	0	0	0	0
455626	338669	R091	0	0	0	0	1	0	0	1	0	0	0	0	0	0	1	0	0	0	0	0	0	1	0	0	0	0	0	0	0
467059	354503	R092	0	0	0	0	1	0	0	1	0	0	0	0	0	0	0	0	1	0	0	1	0	0	0	1	1	0	1	1	0
454423	363686	R093	0	0	0	0	1	0	0	1	0	0	0	0	0	0	1	0	0	0	0	1	0	0	0	1	0	0	1	0	0
456977	379061	R094	0	0	0	0	1	0	1	0	1	0	0	0	1	0	1	0	0	0	1	1	1	0	0	0	0	0	0	0	0
399725	352981	R095	0	0	0	0	0	0	0	1	0	0	0	0	0	0	1	0	0	0	0	0	0	0	0	1	0	0	1	1	0
410738	322043	R096	0	1	0	1	0	0	1	1	0	0	0	0	0	0	1	1	0	0	0	0	1	0	0	0	0	0	0	0	0
409375	345724	R097	0	1	1	0	1	0	0	1	0	0	0	0	0	0	1	1	0	0	0	1	0	0	0	0	0	0	1	0	0
415151	343022	R098	1	1	0	1	0	1	1	1	1	1	0	0	0	0	1	1	0	0	1	1	1	0	0	0	0	0	1	0	0
428122	351663	R099	0	1	0	0	1	1	0	1	1	0	0	0	0	0	1	1	0	0	0	0	1	0	0	0	0	1	1	0	0
403548	371230	R100	0	1	0	1	0	0	0	1	0	0	0	0	0	0	1	0	0	0	0	1	0	0	0	1	1	0	0	1	0
402906	371211	R101	0	0	0	0	0	0	0	1	0	0	0	0	0	0	0	0	0	0	0	1	0	0	0	1	1	0	0	0	0
402375	371488	R102	0	0	0	0	0	0	0	0	0	0	0	0	0	0	0	0	0	0	0	0	0	0	0	1	1	0	0	0	0
401687	370833	R103	0	0	0	0	0	0	0	0	0	0	0	0	0	0	0	0	0	0	0	0	0	0	0	1	1	0	0	0	0
401162	370349	R104	0	0	0	0	0	0	0	0	0	0	0	0	0	0	0	0	0	0	0	0	0	0	0	1	1	0	0	0	0
411502	366609	R105	0	1	0	1	1	0	0	0	0	0	0	0	0	0	0	0	0	0	0	1	0	0	0	1	1	0	1	0	0
410693	382462	R106	0	0	0	0	0	0	0	1	0	0	0	0	1	0	1	0	1	0	1	0	0	0	0	1	0	0	0	1	0
415026	391436	R107	0	1	0	0	1	1	0	1	1	0	0	0	1	0	1	0	0	0	0	1	0	0	0	0	0	0	1	0	0
429433	397740	R108	0	0	0	0	0	0	0	1	1	0	1	0	1	0	1	0	0	0	0	0	1	0	1	0	0	0	0	0	0
406373	393839	R109	0	1	0	0	0	0	1	1	0	1	0	0	0	0	1	0	0	0	0	0	1	0	0	0	0	0	0	0	0
404976	401489	R110	0	1	0	1	1	0	1	1	0	1	0	0	0	1	1	1	0	1	1	1	1	0	1	0	0	0	0	0	0
411790	405687	R111	0	0	0	0	0	1	1	0	0	1	0	0	0	1	1	0	0	0	1	0	1	0	1	0	0	0	0	0	0
407564	408579	R112	0	0	0	0	0	1	1	0	0	0	0	0	0	1	1	0	0	1	0	0	1	0	1	0	0	0	0	0	0
393071	257180	R113	0	0	1	0	1	0	1	1	0	1	0	0	0	0	1	0	0	0	0	1	1	0	0	0	0	0	0	0	0
400543	268707	R114	1	0	0	0	0	0	1	1	0	1	0	0	1	1	1	0	0	0	0	1	1	1	0	0	0	0	0	0	0
395209	274443	R115	0	1	0	0	1	0	1	1	1	1	0	0	0	1	1	1	0	1	0	0	1	0	1	0	0	0	0	0	0
400930	286376	R116	0	0	0	0	1	0	1	1	1	1	0	0	0	1	1	0	0	0	0	1	1	0	1	0	0	1	0	1	0
342683	222031	R117	0	0	0	0	0	0	1	1	0	0	0	0	0	0	1	1	0	0	0	1	1	0	0	0	0	0	0	0	0
355396	249125	R118	1	0	0	0	0	1	1	1	0	1	0	0	0	0	1	0	0	0	1	1	1	0	0	0	0	0	0	0	0
364691	269379	R119	0	0	0	0	0	0	0	1	0	0	0	0	0	0	1	0	0	0	0	1	1	0	0	0	0	0	0	0	0
368922	247110	R120	0	0	0	0	0	0	1	0	0	0	0	0	0	0	1	0	0	0	0	1	1	0	0	0	0	0	0	0	0
383901	265829	R121	0	0	1	0	1	0	1	1	0	0	0	0	0	0	1	0	0	0	0	1	1	0	0	0	0	0	0	0	0

371643	280386	R122	0	0	1	1	1	0	0	1	0	0	0	0	0	0	1	1	0	0	0	1	1	0	0	0	0	0	0	0	0
389599	304287	R123	1	0	1	0	0	1	1	1	0	0	1	0	0	0	1	0	0	1	1	1	1	0	0	0	0	0	0	0	0
379956	305342	R124	0	0	0	0	1	0	0	1	0	0	0	0	0	0	1	0	0	0	0	0	1	0	0	0	0	0	0	0	0
380639	314304	R125	0	0	1	0	0	0	0	1	0	0	0	0	0	0	1	0	0	0	0	1	1	0	0	0	0	1	0	0	0
368333	321583	R126	0	0	0	0	0	0	0	1	0	0	0	0	0	0	1	0	1	0	0	0	0	0	0	0	0	0	1	0	0
385705	329160	R127	0	0	0	0	0	0	0	0	0	0	0	0	0	0	1	0	0	0	0	1	0	0	0	0	0	0	1	0	0
385264	330139	R128	0	0	0	0	0	0	0	0	0	0	0	0	0	0	0	0	0	0	0	1	0	0	0	1	1	0	1	0	0
385246	330984	R129	0	0	0	0	0	0	0	1	0	0	0	0	0	0	0	0	0	0	0	0	0	0	0	1	1	0	0	0	0
384867	331603	R130	0	0	0	0	0	0	0	0	0	0	0	0	0	0	0	0	1	0	0	0	0	0	0	1	1	0	0	0	0
339320	250926	R131	0	0	0	0	1	0	1	1	0	0	0	0	0	0	1	0	0	0	0	1	1	0	0	0	0	0	0	0	0
340295	266446	R132	0	0	0	0	1	0	1	1	0	0	0	0	0	0	1	0	0	0	0	1	1	0	0	0	0	0	0	0	0
351930	266746	R133	0	0	0	0	0	0	1	1	0	0	0	0	0	0	1	0	0	0	0	0	1	0	0	0	0	0	0	0	0
361608	292150	R134	0	1	0	1	0	0	0	1	0	0	0	0	0	1	1	1	0	0	0	1	1	1	0	0	0	0	0	0	0
323965	260284	R135	0	0	0	0	1	1	1	0	0	1	0	1	1	0	1	0	0	0	1	1	1	0	0	0	0	0	0	0	1
310260	257789	R136	0	0	0	0	0	0	1	0	0	0	0	1	0	0	1	0	0	0	0	1	1	1	1	0	0	0	0	0	1
309016	264890	R137	0	0	1	0	0	0	0	0	0	0	0	0	0	0	1	0	0	1	0	1	1	0	0	0	0	0	0	0	0
299836	279699	R138	1	1	0	0	0	0	1	1	1	0	0	0	0	0	1	0	0	0	1	1	1	0	0	0	0	0	0	0	1
284374	266225	R139	0	0	0	0	0	0	0	1	0	0	0	0	0	0	1	0	0	0	0	0	1	0	0	0	0	0	0	0	0
292090	255742	R140	0	0	1	0	1	0	1	0	0	0	0	0	0	0	1	0	0	0	0	1	1	0	0	0	0	0	0	0	1
285680	285959	R141	0	0	0	0	1	1	0	1	1	0	0	0	0	0	1	0	0	0	0	0	1	0	0	0	0	0	0	0	0
363449	134558	R142	0	0	0	0	0	0	0	0	0	1	0	0	0	0	1	0	0	0	0	1	1	0	0	0	0	0	0	0	1
368601	143386	R143	0	1	0	0	1	0	1	1	0	0	0	0	0	0	0	0	0	0	0	1	1	0	0	0	0	0	0	0	0
379930	163542	R144	1	0	0	0	0	0	0	0	0	0	0	0	1	0	0	0	0	1	0	0	0	0	0	0	0	0	0	0	0
381129	150765	R145	0	0	0	0	1	0	1	1	0	0	0	0	0	0	1	0	0	0	1	1	1	0	0	0	0	0	0	0	0
371283	173851	R146	0	0	0	0	1	0	0	1	0	0	0	0	0	0	1	0	0	0	0	0	1	0	0	0	0	0	0	0	0
369645	158552	R147	0	0	0	0	1	0	1	1	0	0	0	0	0	0	1	1	0	1	0	0	1	0	1	0	0	0	0	0	0
374561	215619	R148	0	1	0	0	1	0	1	1	0	0	0	0	0	0	1	1	0	0	0	0	1	0	0	0	0	0	0	0	0
349966	137816	R149	0	1	0	0	0	0	1	1	0	1	0	0	0	1	1	1	0	0	0	0	1	0	0	0	0	0	0	0	0
350877	166219	R150	0	0	0	0	0	0	0	1	0	1	0	0	0	0	1	0	0	0	0	0	1	0	1	0	0	0	0	0	0
353139	177532	R151	0	0	0	0	0	0	1	0	0	0	0	0	0	0	0	0	0	0	0	1	1	0	0	0	0	0	0	0	0
343259	210392	R152	0	0	0	0	1	0	1	0	0	0	0	0	0	0	1	0	0	0	0	0	1	0	0	0	0	0	0	0	1
364499	189507	R153	0	0	0	0	0	0	1	0	0	0	0	0	0	0	0	0	0	0	0	0	1	0	0	0	0	0	0	0	0
357737	214675	R154	0	0	0	0	1	0	1	1	0	0	0	0	0	0	1	1	0	0	0	1	1	0	0	0	0	0	0	0	1
363550	225248	R155	0	0	0	0	1	0	1	1	0	0	0	1	0	0	1	0	0	1	0	0	1	0	0	0	0	0	0	0	0
372208	196388	R156	0	0	0	0	1	0	1	0	0	0	0	0	0	0	1	0	0	0	0	0	1	0	0	0	0	0	0	0	0
332667	142459	R157	0	0	0	0	0	0	0	0	0	0	0	0	0	0	0	0	0	1	0	1	1	0	0	0	0	0	0	0	0
344338	158737	R158	0	0	0	0	1	0	1	1	0	1	0	0	0	1	1	1	0	0	0	1	1	0	0	0	0	0	0	0	0
335264	187707	R159	1	1	0	0	0	0	1	1	0	0	0	0	0	0	1	1	0	0	0	1	1	0	0	0	0	0	0	0	0
332931	174579	R160	0	0	0	0	1	0	1	1	0	0	0	0	0	0	1	1	0	0	0	1	1	0	0	0	0	0	0	0	0
325098	191003	R161	0	0	0	0	1	0	0	0	0	0	0	0	0	0	0	0	0	0	0	1	0	0	0	0	0	0	0	0	0
304778	171016	R162	0	0	0	1	1	0	1	1	0	1	0	0	0	0	1	0	0	0	1	1	1	1	0	0	0	0	0	0	0

294038	178340	R163	0	0	0	0	1	0	1	1	0	0	0	0	0	1	1	0	0	0	0	0	1	0	0	0	0	0	0	0	0
314547	203304	R164	0	1	0	1	1	0	1	1	0	0	0	0	0	0	1	1	0	1	0	1	1	0	0	0	0	0	0	0	1
298697	190893	R165	0	0	0	0	0	0	1	1	0	0	0	0	0	0	1	0	0	1	0	0	1	0	1	0	0	0	0	0	0
304101	210194	R166	0	0	0	0	1	0	1	0	0	0	0	0	0	0	1	0	0	0	0	0	1	0	0	0	0	0	0	0	0
336415	236843	R167	0	0	0	0	1	1	1	1	0	0	0	1	0	0	1	0	0	0	0	0	1	0	0	0	0	0	0	0	1
319171	215737	R168	0	0	0	0	1	0	1	1	0	0	0	0	0	0	1	0	0	0	0	1	0	0	0	0	0	0	0	0	0
329194	218645	R169	0	1	0	0	0	0	1	0	0	0	0	0	0	0	0	0	0	0	0	1	1	0	0	0	0	0	0	0	0
290080	211916	R170	1	0	0	0	0	0	1	0	0	0	0	0	0	0	1	1	0	0	0	0	1	0	0	0	0	0	0	0	0
234525	162477	R171	0	0	0	0	0	0	1	0	0	0	0	0	0	0	1	0	0	1	1	0	1	0	0	0	0	0	0	0	0
248945	200279	R172	0	0	0	0	1	0	1	1	0	1	0	0	0	0	1	1	0	1	0	0	1	0	0	0	0	0	0	0	1
235135	184302	R173	0	0	0	0	1	0	1	1	0	0	0	1	0	0	1	0	0	0	0	0	1	0	0	0	0	0	0	0	0
269405	207580	R174	0	0	0	0	0	0	1	0	0	0	0	0	0	0	1	0	0	0	0	0	1	0	0	0	0	0	0	0	0
266168	250921	R175	0	0	0	0	0	1	0	1	0	0	0	0	1	0	1	0	0	0	0	0	1	0	0	0	0	0	0	0	0
266628	217690	R176	0	1	0	0	1	0	1	1	0	0	0	0	0	0	1	0	0	0	0	1	1	0	0	0	0	0	0	0	0
252398	237302	R177	0	0	0	0	1	0	1	1	0	0	0	0	0	0	1	0	0	0	0	0	1	0	1	0	0	0	0	0	0
265802	266224	R178	0	0	0	0	1	1	0	1	0	0	0	0	0	0	1	0	0	0	0	0	1	0	0	1	1	0	1	0	0
264381	267931	R179	0	0	0	0	0	0	0	0	0	0	0	0	0	0	1	0	0	0	0	1	0	0	0	1	1	0	1	0	0
263691	268282	R180	0	0	0	0	1	0	0	0	0	0	0	0	0	0	0	0	0	0	0	0	0	0	0	1	1	0	1	0	0
263355	267487	R181	0	0	0	0	0	1	0	1	0	0	0	0	0	0	0	0	0	0	0	0	0	0	0	1	0	0	1	0	0
286593	275744	R182	0	0	1	1	1	1	0	1	0	0	0	0	0	0	1	0	0	0	0	1	0	0	0	1	0	0	0	0	0
285824	275170	R183	0	0	0	0	0	0	0	1	0	0	0	0	0	0	1	0	0	0	0	0	0	0	0	0	0	0	0	0	0
285354	275669	R184	0	0	0	0	0	0	0	1	0	0	0	0	0	0	0	0	0	0	0	1	0	0	0	1	0	0	1	0	0
285028	276150	R185	0	0	0	0	0	0	0	0	1	0	0	0	0	0	0	0	0	0	0	1	0	0	0	1	0	0	1	0	0
232742	171093	R186	0	0	0	1	0	0	1	1	0	0	0	1	0	0	1	0	0	0	0	0	1	0	0	0	0	0	0	0	0
222812	162350	R187	0	0	0	0	1	0	0	1	0	0	0	0	0	0	1	0	0	1	0	0	1	0	0	0	0	1	0	0	0
208850	172142	R188	0	0	0	0	1	0	0	0	0	0	0	0	1	0	1	0	0	1	0	1	1	0	0	0	0	0	0	0	0
220273	193519	R189	1	0	1	1	0	0	0	1	0	0	0	0	0	0	1	0	0	0	0	1	1	0	0	0	0	0	0	0	0
239109	207983	R190	0	1	0	1	1	0	1	1	0	0	0	0	0	1	1	0	0	0	0	1	1	0	0	0	0	1	0	0	0
249961	213903	R191	0	1	0	1	0	0	1	1	0	1	0	0	1	0	1	0	0	0	1	1	1	0	0	0	0	0	0	0	1
233806	234287	R192	0	0	0	0	0	0	1	1	0	1	0	0	0	0	1	0	0	0	1	1	1	0	0	0	0	0	0	0	0
291719	230602	R193	0	0	1	0	1	0	0	0	0	0	0	0	0	0	0	0	0	0	0	1	1	0	0	0	0	0	0	0	0
247314	259218	R194	0	0	0	0	1	1	0	0	1	0	0	0	0	0	1	0	0	0	0	0	1	0	0	0	0	0	0	0	0
190484	156488	R195	0	0	0	0	1	0	1	0	0	0	0	0	0	0	1	0	0	0	0	0	1	0	0	0	0	0	0	0	0
226421	210040	R196	0	0	0	0	0	0	0	0	0	0	0	0	0	0	1	0	0	0	0	1	1	1	0	0	0	0	0	0	0
203544	198467	R197	0	0	0	0	1	0	1	1	0	1	0	1	0	0	1	0	0	1	1	0	1	0	1	0	0	0	0	0	1
201502	188618	R198	0	0	1	1	1	0	1	1	0	0	0	0	0	0	1	1	0	1	0	1	1	0	0	0	0	0	0	0	0
202877	221967	R199	0	0	0	0	0	0	1	0	1	0	0	0	0	0	1	0	0	0	1	0	1	0	0	0	0	0	0	0	0
220527	221258	R200	0	0	0	0	0	0	0	1	0	1	0	0	0	0	1	0	0	0	0	1	1	0	0	0	0	0	0	0	0
183096	241833	R201	0	0	0	0	0	0	0	0	1	0	0	0	0	0	1	0	0	1	1	0	1	0	0	0	0	0	0	0	0
188962	197594	R202	0	0	0	0	1	0	1	1	0	1	0	0	1	0	1	0	0	1	0	1	1	0	1	0	0	0	0	0	0
179662	222289	R203	0	1	0	0	0	0	0	1	0	0	1	0	0	0	1	0	0	1	0	1	1	0	1	0	0	0	0	0	1

168380	209071	R204	0	0	0	0	0	0	1	0	0	0	0	0	1	0	1	0	0	0	0	0	1	0	1	0	0	0	0	0	0
172116	238235	R205	0	0	1	0	1	1	1	1	0	0	0	1	0	0	1	0	0	0	1	0	1	0	0	0	0	1	0	0	0
167458	223929	R206	0	0	0	0	1	0	0	1	0	1	0	1	0	0	1	0	0	0	1	0	1	0	0	0	0	0	0	0	0
147369	193909	R207	0	0	0	0	0	0	1	1	0	0	0	0	0	0	1	0	0	1	0	1	1	0	0	0	0	0	0	0	1
147558	231063	R208	0	0	0	0	0	0	1	1	0	0	0	0	0	0	1	0	0	0	1	0	1	0	0	0	0	0	0	0	1
144401	205081	R209	0	0	0	0	1	0	1	1	0	0	0	0	0	0	1	0	0	0	0	1	1	0	0	0	0	0	0	0	0
125440	226866	R210	0	0	0	0	0	0	1	0	0	0	0	0	0	0	1	0	0	1	0	0	1	0	0	0	0	0	0	0	0
147632	255883	R211	0	0	0	0	0	0	1	0	0	0	0	0	0	0	1	0	0	1	0	0	1	0	0	0	0	0	0	0	0
163286	255323	R212	0	0	0	0	0	0	0	0	0	1	0	0	0	0	1	0	0	1	0	0	1	0	0	0	0	0	0	0	0
158307	247787	R213	0	0	0	0	0	0	1	0	0	0	0	0	0	0	1	0	0	0	0	0	1	0	0	0	0	0	0	0	0
157009	148878	R214	0	0	0	0	0	0	1	1	0	1	0	0	0	0	1	0	0	0	0	0	1	0	1	0	0	0	0	0	1
174493	157346	R215	0	0	0	0	0	0	1	1	0	1	0	0	0	0	1	0	0	0	0	0	1	0	0	0	0	0	0	0	0
141522	174983	R216	0	0	0	0	0	0	1	1	0	1	0	0	0	0	1	0	0	1	0	1	1	0	0	0	0	0	0	0	0
107952	136304	R217	0	1	1	1	1	0	1	1	0	1	0	0	0	1	1	1	0	1	0	1	1	0	0	0	0	0	0	0	0
129377	143764	R218	0	0	0	0	0	0	1	0	0	1	0	0	0	0	1	0	0	1	0	0	1	0	0	0	0	0	0	0	0
172554	173869	R219	0	0	0	0	1	0	1	1	0	1	0	0	0	0	1	0	0	0	0	1	1	0	0	0	0	0	0	0	1
133889	183439	R220	0	0	0	0	0	1	1	1	0	1	0	0	0	0	1	0	0	1	0	0	1	0	1	0	0	0	0	0	1
116614	181368	R221	0	0	0	0	0	0	1	0	0	0	0	0	0	0	0	0	0	1	0	1	1	0	0	0	0	0	0	0	0
134701	159838	R222	0	0	0	0	0	0	0	0	0	1	0	0	0	0	1	0	0	0	0	0	1	0	0	0	0	0	0	0	0
97239	168926	R223	0	0	0	0	0	0	1	1	0	1	0	0	0	0	1	0	0	1	0	0	1	0	0	0	0	0	0	0	1
357520	322617	R224	0	0	1	0	1	0	1	0	1	0	1	0	0	0	1	0	0	0	0	0	1	0	0	0	0	0	0	0	0
386041	354956	R225	1	0	0	0	0	0	0	0	1	0	0	0	0	0	1	0	0	0	0	1	1	0	0	0	0	0	0	0	0
389175	369802	R226	0	0	0	1	1	1	0	1	0	0	0	0	0	0	1	0	0	0	1	1	0	0	0	1	0	0	1	1	0
383802	381241	R227	0	0	1	1	0	1	1	1	1	0	0	0	0	0	1	0	0	0	0	1	1	0	0	0	0	0	0	0	0
358446	388774	R228	0	0	0	0	1	0	0	1	0	0	0	0	0	0	1	1	0	0	0	1	1	0	0	0	0	0	0	0	0
384810	402755	R229	0	0	0	0	1	0	1	0	0	0	1	0	0	0	1	0	0	0	0	0	0	1	0	0	0	0	0	0	0
354091	307650	R230	0	0	0	0	1	1	1	1	0	1	0	0	0	1	1	0	0	0	0	0	0	0	0	0	0	0	0	0	0
354837	342155	R231	1	1	1	0	0	0	1	0	0	1	1	0	0	1	1	1	0	0	0	1	1	0	0	0	0	0	0	0	0
345310	349968	R232	0	0	0	0	0	0	1	0	0	0	0	1	1	0	1	0	0	0	1	0	1	0	1	0	0	0	0	0	0
363681	354411	R233	1	0	0	0	0	1	1	0	0	0	0	0	0	0	1	0	0	0	0	0	1	0	0	0	0	0	0	0	0
379377	347675	R234	0	1	0	0	1	0	1	1	0	0	0	0	0	1	1	0	0	0	0	0	0	0	0	0	0	0	0	0	0
363159	373975	R235	0	0	0	0	1	0	0	1	0	0	0	0	0	0	1	0	0	0	0	1	1	0	0	0	0	0	0	0	0
374719	368478	R236	0	0	0	0	1	1	0	0	0	0	0	0	0	0	1	0	0	0	1	1	1	0	1	1	0	0	0	0	0
364340	405504	R237	0	0	0	0	1	0	1	1	0	1	0	0	0	0	1	0	0	0	1	1	0	0	0	0	0	0	1	0	0
348704	386475	R238	0	0	0	0	0	0	1	1	0	0	0	0	0	0	1	0	0	0	0	1	1	0	0	0	0	0	0	0	0
366027	398291	R239	0	0	1	0	0	0	0	1	1	0	0	0	0	0	1	0	0	0	0	0	0	0	0	0	0	0	1	0	0
317728	313093	R240	0	0	0	0	0	0	1	0	0	0	0	0	0	1	1	0	0	0	0	0	1	0	0	0	0	0	0	0	0
337776	323275	R241	0	0	0	0	0	0	1	0	0	0	1	0	0	0	1	1	0	0	0	0	1	0	0	0	0	0	0	0	0
327358	348726	R242	0	0	0	0	0	0	1	0	0	0	0	1	0	1	1	0	0	0	1	0	1	0	1	0	0	0	0	0	0
346679	414283	R243	0	0	0	0	1	0	1	0	0	0	1	0	0	0	1	0	0	0	0	0	1	0	0	0	0	0	0	0	0
303574	307155	R244	0	0	0	0	1	0	1	0	0	0	0	0	0	0	1	0	0	0	0	0	1	0	0	0	0	0	0	0	0

337788	291848	R245	0	0	1	1	0	1	1	0	0	0	0	0	0	1	1	1	0	0	1	0	1	0	1	0	0	0	0	0	0
309668	324959	R246	0	0	0	0	0	0	1	0	0	0	0	0	0	1	1	0	0	0	1	0	1	0	1	0	0	0	0	0	0
312191	355241	R247	0	0	0	0	1	0	1	0	0	0	0	0	0	0	1	0	0	0	0	0	1	0	1	0	0	0	0	0	0
325102	371433	R248	1	1	1	0	1	1	1	0	0	1	0	0	0	1	1	1	0	1	1	1	1	0	0	0	0	1	0	0	0
328546	386742	R249	0	0	0	0	1	0	0	1	1	0	0	1	0	1	1	0	0	0	0	0	1	0	0	0	0	0	0	0	0
340224	372178	R250	0	0	0	0	1	1	1	0	1	0	0	0	0	0	1	0	0	0	0	0	1	0	0	0	0	0	0	0	0
337842	397674	R251	0	1	0	1	0	1	1	1	0	0	0	0	0	0	1	1	0	0	0	1	1	0	0	0	0	0	0	0	0
322464	409140	R252	0	0	1	1	0	0	1	1	0	0	1	0	0	0	1	1	0	0	0	1	1	1	0	0	0	0	0	0	0
347989	400177	R253	0	0	1	0	1	1	0	1	0	0	0	0	0	0	1	0	0	0	0	1	1	0	0	0	0	1	1	0	0
284133	304950	R254	0	0	0	0	1	0	1	1	0	0	0	0	0	1	1	1	0	0	0	0	1	0	1	0	0	0	0	0	0
290674	317804	R255	0	0	1	0	0	0	1	0	0	0	0	0	0	0	1	0	0	0	0	1	1	0	0	0	0	0	0	0	0
304333	339446	R256	0	0	0	1	0	1	1	0	0	1	0	1	0	1	1	0	0	0	1	0	1	0	0	0	0	0	0	0	0
287515	380231	R257	0	1	1	1	1	1	1	1	0	1	0	0	0	1	1	1	0	0	1	1	1	0	0	0	0	0	0	0	0
316630	377976	R258	1	0	0	0	1	0	0	0	0	1	1	0	1	0	1	1	0	0	0	1	1	0	0	0	0	0	0	0	0
307027	409073	R259	0	0	0	0	1	0	1	0	0	1	0	0	0	1	1	1	0	0	1	0	1	0	1	0	0	0	0	0	0
269183	307030	R260	0	0	0	0	1	1	1	1	0	1	0	0	0	1	1	0	0	0	1	0	1	0	0	0	0	0	0	0	0
281118	343321	R261	0	0	1	0	1	0	1	1	0	0	0	0	0	1	1	0	0	0	0	1	1	0	1	0	0	0	0	0	1
257450	299808	R262	0	1	1	0	1	1	0	1	0	0	0	0	0	1	1	1	0	0	1	1	1	0	0	1	0	0	0	0	0
258031	300529	R263	0	0	0	0	1	0	0	1	0	0	0	0	0	0	1	0	0	0	0	1	0	0	0	1	0	0	1	0	0
258439	300861	R264	0	0	0	1	0	0	0	1	0	0	0	0	0	0	1	0	1	0	0	1	0	0	0	1	0	0	1	0	0
258760	301138	R265	0	0	1	0	0	1	0	1	0	0	0	0	0	0	1	0	0	0	0	1	0	0	0	1	0	0	1	0	0
255340	298822	R266	0	0	0	0	1	1	0	0	0	0	0	0	0	0	0	0	1	0	0	0	0	0	0	0	1	0	0	1	0
281347	330108	R267	0	0	0	0	0	1	1	1	1	1	0	1	0	0	1	0	0	0	1	0	1	0	1	0	0	0	0	0	0
255460	317475	R268	0	0	0	0	1	0	1	0	0	0	1	0	0	0	1	0	0	0	0	1	1	0	0	0	0	0	0	0	0
262187	340968	R269	0	1	0	0	1	1	1	0	0	0	0	0	0	0	1	0	0	0	1	0	1	0	1	0	0	0	0	0	0
267986	344200	R270	0	1	0	0	0	0	1	0	0	1	0	0	0	0	1	0	0	0	0	0	1	0	0	0	0	0	0	0	0
277675	412162	R271	0	0	0	0	1	1	1	0	0	0	0	0	0	0	1	1	0	0	0	1	1	0	1	0	0	0	0	0	1
278471	399531	R272	0	1	0	0	1	0	1	0	0	1	0	0	0	1	1	0	0	0	0	0	1	0	1	0	0	0	0	0	0
239651	378287	R273	0	1	1	0	0	0	0	0	0	0	0	0	0	1	1	1	0	0	0	1	1	0	0	0	0	0	0	0	0
236693	378383	R274	0	0	0	0	1	0	0	1	0	0	0	0	0	0	1	0	1	0	0	1	1	0	0	0	0	0	0	0	0
236714	377868	R275	0	0	0	0	1	0	0	0	0	0	0	0	0	0	1	0	1	0	0	1	0	0	0	1	0	0	1	0	0
237183	377283	R276	0	1	0	0	1	1	0	0	0	0	0	0	0	0	0	0	0	0	0	0	0	0	0	1	1	0	0	0	0
256651	411492	R277	0	0	1	1	1	0	1	1	0	0	1	0	0	1	1	0	0	0	0	1	1	0	0	0	0	0	0	0	1
257673	283205	R278	0	0	0	0	1	1	1	1	0	1	0	0	0	1	1	0	0	0	0	1	1	0	0	0	0	0	0	0	0
239921	262303	R279	0	0	1	0	0	0	1	0	0	0	0	0	0	1	1	0	0	0	0	1	1	0	1	0	0	0	0	0	0
225686	272840	R280	0	0	0	0	0	0	1	1	0	1	1	0	0	0	1	0	0	0	0	0	1	0	1	0	0	0	0	0	0
248555	273354	R281	0	0	0	1	1	0	1	1	1	1	0	0	0	1	1	0	0	0	1	0	1	0	1	0	0	0	0	0	0
230178	296720	R282	0	0	0	0	0	1	1	1	0	1	0	0	0	1	1	0	0	0	0	0	1	0	1	0	0	0	0	0	1
213758	292502	R283	0	0	0	0	1	0	1	1	0	1	0	0	0	1	1	0	0	1	1	0	1	0	0	0	0	0	0	0	0
239651	313332	R284	0	0	0	0	1	1	1	0	0	1	0	1	1	1	1	0	0	0	0	0	1	0	0	0	0	0	0	0	1
227941	337264	R285	0	1	0	0	0	0	1	0	0	1	0	0	0	0	1	0	0	0	0	1	1	0	1	0	0	0	0	0	0

240941	353512	R286	0	0	0	0	1	0	1	1	0	0	0	0	0	1	1	1	0	0	0	0	1	0	1	0	0	0	0	0	0
258125	372673	R287	0	0	1	0	0	0	1	1	0	1	0	0	0	1	1	1	0	0	0	0	1	0	1	0	0	0	0	0	1
262488	380240	R288	0	1	0	0	0	0	1	0	0	0	0	0	0	1	1	0	0	0	0	0	1	0	0	0	0	0	0	0	1
225363	387817	R289	0	0	0	0	1	0	1	1	0	1	0	0	0	0	1	1	0	0	1	1	1	0	0	0	0	0	0	0	0
233733	409466	R290	0	0	0	0	1	1	1	0	0	1	0	0	0	0	1	0	0	0	0	0	1	0	1	0	0	0	0	0	0
224437	418718	R291	0	0	0	0	1	0	1	1	0	0	0	0	0	1	1	0	0	0	0	0	1	0	0	0	0	0	0	0	0
201663	267341	R292	0	0	0	0	0	1	0	1	1	0	0	0	0	1	1	1	0	0	0	1	1	0	0	0	0	0	0	0	0
202257	267241	R293	0	0	0	1	0	0	0	1	1	0	0	0	0	0	1	0	0	0	0	1	0	0	0	0	0	0	1	0	0
202706	267190	R294	0	0	0	0	0	0	0	1	0	0	0	0	0	0	0	0	0	0	0	1	0	0	0	1	1	0	0	0	0
203400	266768	R295	0	0	0	0	0	0	0	0	0	0	0	0	0	0	0	0	0	0	0	0	0	0	0	1	0	0	1	0	0
203855	266635	R296	0	0	0	0	0	1	0	0	1	0	0	0	0	0	0	0	0	0	0	0	0	1	0	1	0	0	1	0	0
204442	266376	R297	0	0	0	0	0	1	0	0	0	0	0	0	0	0	0	0	0	0	0	0	0	0	0	0	1	0	0	0	0
216954	282333	R298	0	0	0	0	1	0	1	1	0	1	0	0	0	1	1	0	0	0	0	0	1	0	1	0	0	0	0	0	0
216298	323949	R299	1	1	0	0	1	0	1	0	0	1	0	0	0	0	1	0	0	0	0	0	1	0	1	0	0	0	0	0	1
207693	317416	R300	1	0	0	0	0	0	1	0	0	1	0	0	0	0	1	0	0	0	0	0	1	0	0	0	0	0	0	0	0
230342	321084	R301	0	0	0	0	0	0	1	1	0	1	1	0	1	1	1	1	0	0	0	1	1	0	1	0	0	0	0	0	0
217126	342529	R302	0	1	0	0	0	0	1	0	0	0	0	0	0	1	1	0	0	0	1	0	1	0	0	0	0	0	0	0	0
200051	355615	R303	0	0	0	0	0	0	1	0	0	0	1	0	0	0	1	0	0	0	0	0	1	0	0	0	0	0	0	0	0
215107	364197	R304	0	1	0	0	0	0	1	0	0	1	1	0	0	1	1	1	0	1	0	1	1	0	0	0	0	0	0	0	0
215901	387735	R305	0	0	0	1	1	0	1	0	0	1	0	0	0	0	1	0	0	0	1	1	1	0	0	0	0	0	0	0	0
267210	395916	R306	0	1	0	0	1	0	1	1	0	1	0	0	0	1	1	0	0	0	0	0	1	0	0	0	0	0	0	0	1
197132	307885	R307	0	0	0	0	0	0	1	0	0	1	0	0	0	0	1	0	0	1	0	0	1	0	1	0	0	0	0	0	1
190833	284239	R308	0	0	0	0	0	1	1	1	0	0	0	0	0	1	1	0	0	0	1	0	1	0	0	0	0	0	0	0	1
202100	289947	R309	0	0	0	0	0	0	1	0	0	0	1	0	0	0	1	0	0	1	1	0	1	0	1	0	0	0	0	0	1
202137	375538	R310	0	0	0	0	1	0	1	0	0	1	0	0	0	1	1	1	0	1	0	0	1	1	0	0	0	0	0	0	1
212815	371407	R311	0	1	1	1	1	0	0	0	0	1	1	0	0	1	1	0	0	0	1	1	1	0	0	0	0	0	0	0	0
179318	279641	R312	0	0	0	0	0	0	1	1	0	1	0	0	0	0	1	0	0	0	1	0	1	0	0	0	0	0	0	0	0
187455	304878	R313	0	0	0	0	0	0	1	0	0	0	0	0	0	0	1	0	0	0	0	0	1	0	0	0	0	0	0	0	0
178788	314750	R314	0	0	1	1	0	0	1	1	0	0	0	0	0	1	1	0	0	0	1	0	1	0	1	0	0	0	0	0	1
177436	314147	R315	1	0	0	0	0	0	1	0	0	1	0	0	0	1	1	0	0	1	1	0	1	0	0	0	0	0	0	0	1
248991	294075	R316	0	0	0	1	0	0	0	1	0	1	0	0	0	0	1	0	0	0	0	1	1	0	0	0	0	0	0	0	0
141147	290116	R317	0	0	0	1	0	0	0	1	0	0	0	0	0	0	1	0	0	0	0	0	1	1	0	0	0	0	0	0	0
164583	326488	R318	0	0	0	0	0	0	1	0	0	0	0	0	0	0	1	0	0	1	0	0	1	0	0	0	0	0	0	0	0
154492	332574	R319	0	0	0	0	0	0	1	0	0	1	0	0	0	1	1	0	0	1	0	0	1	0	0	0	0	0	0	0	0
145102	349757	R320	0	0	0	0	1	0	1	1	0	1	1	0	0	0	1	1	0	1	0	0	1	1	0	0	0	0	0	0	0
154745	343993	R321	0	0	1	0	0	0	1	0	0	1	0	0	0	1	1	0	0	1	0	0	1	0	0	0	0	0	0	0	1
188512	387673	R322	0	0	0	0	1	0	1	1	0	1	0	0	0	1	1	1	0	0	0	0	1	0	0	0	0	0	0	0	0
185853	391315	R323	0	0	0	0	1	0	1	1	0	1	0	0	0	1	1	0	0	1	0	1	1	0	1	0	0	0	0	0	0
406547	209059	R324	0	1	0	1	0	0	1	1	0	0	0	0	0	1	1	1	1	0	0	1	1	0	0	0	0	0	0	0	0
395414	194103	R325	0	0	0	0	0	0	1	1	0	1	0	0	0	1	1	1	0	0	0	0	1	0	0	0	0	0	0	0	0
446534	291614	R326	0	0	1	0	1	1	1	1	0	0	0	0	1	0	1	0	0	0	0	0	1	0	0	0	0	0	0	0	0

446677	307650	R327	0	0	0	0	1	0	0	1	0	0	0	0	0	0	1	0	0	0	1	0	1	0	0	0	0	0	0	0	0
437919	297464	R328	0	1	1	0	1	0	0	1	1	0	0	0	0	0	1	0	0	0	1	0	1	0	1	0	0	1	0	0	0
446302	328745	R329	0	1	0	0	1	1	0	1	0	1	0	0	0	0	1	0	0	0	0	1	1	0	1	0	0	0	0	0	0
430790	359147	R330	0	0	0	0	1	0	0	1	1	0	0	0	0	0	1	0	1	0	0	0	0	0	0	0	0	0	1	0	0
429962	361947	R331	0	0	0	0	0	0	0	1	1	0	0	0	0	0	1	0	0	0	0	0	0	0	0	0	0	0	1	0	0
429819	371340	R332	0	0	0	0	0	1	0	1	0	0	0	0	0	1	1	0	0	0	0	0	0	0	0	0	0	0	0	0	0
433413	378158	R333	0	0	0	0	1	0	0	0	0	0	0	0	0	0	0	0	0	0	0	0	0	0	0	1	0	0	0	1	0
514933	340351	R334	0	1	0	0	1	1	0	1	0	0	0	0	0	1	1	0	0	0	0	0	1	0	1	0	0	0	0	0	0
377773	414085	R335	0	0	0	0	0	1	0	0	0	1	0	0	0	0	1	0	0	0	1	0	1	0	1	0	0	0	0	0	0

**Acronyms**	**Species name**
**sp1**	***Polyrhachis lamellidens***
**sp2**	***Myrmecina nipponica***
**sp3**	***Camponotus kiusuensis***
**sp4**	***Ponera scabra***
**sp5**	***Lasius japonicus***
**sp6**	***Formica japonica***
**sp7**	***Pristomyrmex pungens***
**sp8**	***Pheidole fervida***
**sp9**	***Mymica carinata***
**sp10**	***Crematogaster osakensis***
**sp11**	***Crematogaster vagula***
**sp12**	***Crematogaster matsumurai***
**sp13**	***Lasius spathepus***
**sp14**	***Strumigenys lewisi***
**sp15**	***Paratrechina flavipes***
**sp16**	***Vollenhovia emeryi***
**sp17**	***Stenamma owstoni***
**sp18**	***Pachycondyla chinensis***
**sp19**	***Camponotus japonicus***
**sp20**	***Aphaenogaster japonica***
**sp21**	***Pachycondyla javana***
**sp22**	***Cryptone sauteri***
**sp23**	***Tetramorium tsushimae***
**sp24**	***Myrmica kotokui***
**sp25**	***Myrmica kurokii***
**sp26**	***Camponotus nipponensis***
**sp27**	***Camponotus atrox***
**sp28**	***Leptothorax sp.1***
**sp29**	***Leptothorax sp.2***
