# Supplementary material for: Modelling Vulnerability and Range Shifts in Ant Communities Responding to Future Global Warming in Temperate Forests
Source: PLoS One. 2016 Aug 9;11(8):e0159795. doi: 10.1371/journal.pone.0159795 (PMC4978472; doi:10.1371/journal.pone.0159795)
Supplement: S2 Table — (PDF) [file pone.0159795.s003.pdf]

**S2 Table.** Summary of altitude and temperature conditions and statistics of linear regressions between the proportion of predicted suitable geographic areas and period. Optimal and tolerance altitude and temperature are estimated using weighted averaging regression models. A linear regression equation was made from the proportion of suitable geographic areas (predicted using general additive models) against period (2000, 2010, 2020, 2030, 2040, 2050, 2060, 2070, and 2080). An increase means that the number of taxa should significantly increase over time, whereas a decrease means that the number of taxa should significantly decrease over time, and stable means there is a nonspecific trend determined for the number of taxa over time.

| | Altitude and temperature | | | | Linear regression | | | | |
|---|---|---|---|---|---|---|---|---|---|
| Species | Altitudinal range (m) | Optimal altitude (m) | Temperature range (ºC) | Optimal temperature (ºC) | *Slope* | *Intercept* | $R^2$ | *P* | *Response* |
| *Aphaenogaster japonica* | 59~673 | 366 | 9.07~12.44 | 10.76 | -0.79 | 1659 | 0.96 | 0.00 | Decrease |
| *Camponotus atrox* | 517~1092 | 804 | 7.34~9.76 | 8.55 | -0.26 | 559 | 0.64 | 0.01 | Decrease |
| *Camponotus japonicas* | 42~335 | 189 | 10.78~12.99 | 11.88 | -0.73 | 1528 | 0.60 | 0.01 | Decrease |
| *Camponotus kiusuensis* | 117~484 | 300 | 9.88~12.45 | 11.17 | -0.47 | 961 | 0.85 | 0.00 | Decrease |
| *Camponotus nipponensis* | 130~408 | 269 | 9.29~12.09 | 10.69 | -0.43 | 891 | 0.44 | 0.05 | Stable |
| *Crematogaster matsumurai* | 62~266 | 164 | 10.84~12.70 | 11.77 | -0.59 | 1227 | 0.56 | 0.02 | Decrease |
| *Crematogaster osakensis* | 44~298 | 171 | 11.04~13.44 | 12.24 | 0.29 | -508 | 0.89 | 0.00 | Increase |
| *Crematogaster vagula* | 44~244 | 144 | 11.07~13.20 | 12.13 | -0.49 | 1028 | 0.48 | 0.04 | Decrease |
| *Cryptone sauteri* | 2~445 | 223 | 9.66~12.77 | 11.22 | 0.18 | -337 | 0.36 | 0.09 | Stable |
| *Formica japonica* | 52~852 | 452 | 7.98~12.53 | 10.25 | -0.28 | 606 | 0.78 | 0.00 | Decrease |
| *Lasius japonicus* | 30~486 | 258 | 9.80~13.04 | 11.42 | -0.67 | 1399 | 0.67 | 0.01 | Decrease |

| | | | | | | | | | |
|---|---|---|---|---|---|---|---|---|---|
| *Lasius spathepus* | 77~615 | 346 | 9.02~12.31 | 10.66 | 0.16 | -263 | 0.06 | 0.52 | Stable |
| *Leptothorax* sp.1 | 255~949 | 602 | 7.40~11.10 | 9.25 | -0.10 | 232 | 0.57 | 0.02 | Decrease |
| *Leptothorax* sp.2 | 39~228 | 133 | 11.71~13.52 | 12.62 | 0.71 | -1397 | 0.88 | 0.00 | Increase |
| *Mymica carinata* | 134~756 | 445 | 8.88~11.79 | 10.33 | -0.49 | 1055 | 0.63 | 0.01 | Decrease |
| *Myrmecina nipponica* | 42~537 | 290 | 9.28~12.66 | 10.97 | 0.08 | -121 | 0.02 | 0.75 | Stable |
| *Myrmica kotokui* | 790~1269 | 1030 | 6.08~8.97 | 7.52 | 0.37 | -729 | 0.72 | 0.00 | Increase |
| *Myrmica kurokii* | 810~1351 | 1080 | 5.34~8.64 | 6.99 | 0.39 | -766 | 0.63 | 0.01 | Increase |
| *Pachycondyla chinensis* | 8~219 | 114 | 11.86~14.01 | 12.93 | -0.01 | 45 | 0.00 | 0.94 | Stable |
| *Pachycondyla javana* | 30~336 | 183 | 10.65~13.32 | 11.98 | -0.08 | 247 | 0.21 | 0.22 | Stable |
| *Paratrechina flavipes* | 31~474 | 253 | 9.97~13.11 | 11.54 | -0.11 | 296 | 0.12 | 0.36 | Stable |
| *Pheidole fervida* | 64~589 | 327 | 9.32~12.45 | 10.88 | -0.66 | 1378 | 0.70 | 0.00 | Decrease |
| *Polyrhachis lamellidens* | 82~377 | 229 | 10.22~12.67 | 11.45 | 0.09 | -115 | 0.06 | 0.51 | Stable |
| *Ponera scabra* | 106~644 | 375 | 9.19~12.72 | 10.95 | -0.24 | 511 | 0.42 | 0.06 | Stable |
| *Pristomyrmex pungens* | 45~268 | 157 | 10.98~13.50 | 12.24 | 0.35 | -640 | 0.93 | 0.00 | Increase |
| *Stenamma owstoni* | 591~1137 | 864 | 6.55~10.45 | 8.50 | -0.04 | 93 | 0.28 | 0.15 | Stable |
| *Strumigenys lewisi* | 32~366 | 199 | 10.51~13.40 | 11.95 | 0.04 | -18 | 0.01 | 0.81 | Stable |
| *Tetramorium tsushimae* | 36~311 | 174 | 10.67~13.35 | 12.01 | 0.02 | -9 | 0.00 | 0.91 | Stable |
| *Vollenhovia emeryi* | 21~410 | 215 | 9.99~12.96 | 11.47 | -0.77 | 1610 | 0.50 | 0.03 | Decrease |
