# Supplementary material for: Modelling Vulnerability and Range Shifts in Ant Communities Responding to Future Global Warming in Temperate Forests
Source: PLoS One. 2016 Aug 9;11(8):e0159795. doi: 10.1371/journal.pone.0159795 (PMC4978472; doi:10.1371/journal.pone.0159795)
Supplement: S3 Table — (PDF) [file pone.0159795.s004.pdf]

S3 Table. Summary of the calibrated generalized additive models (GAM; corrected by spatial autocorrelation) and the indicators of model accuracy. AUC means area under the curve of receiver operating characteristic.

Species	GAM						Accuracy indicator		
	Nr. Sample	Coefficient	Standard error	Deviance explained (%)	R^2	*P*	AUC	Sensitivity	Specificity
Aphaenogaster japonica	160	1.06	0.08	25.40	0.18	<0.01	0.77	0.66	0.67
Camponotus atrox	52	0.19	0.02	58.00	0.51	<0.01	0.96	0.58	1.00
Camponotus japonicus	67	0.31	0.04	20.50	0.14	<0.01	0.81	0.60	0.82
Camponotus kiusuensis	50	0.15	0.02	13.50	0.09	<0.01	0.79	0.55	0.76
Camponotus nipponensis	18	0.04	0.01	10.80	0.07	<0.01	0.87	0.52	0.95
Crematogaster matsumurai	17	0.06	0.02	11.50	0.06	<0.01	0.90	0.53	1.00
Crematogaster osakensis	95	0.42	0.05	13.80	0.11	<0.01	0.78	0.63	0.82
Crematogaster vagula	27	0.11	0.02	24.30	0.18	<0.01	0.87	0.55	0.95
Cryptone sauteri	32	0.13	0.02	46.20	0.36	<0.01	0.92	0.56	0.93
Formica japonica	77	0.36	0.05	25.80	0.19	<0.01	0.77	0.59	0.80
Lasius japonicus	161	0.95	0.08	23.90	0.14	<0.01	0.74	0.69	0.70
Lasius spathepus	33	0.24	0.06	5.76	0.03	<0.01	0.70	0.53	0.78
Leptothorax sp.1	25	0.11	0.02	38.90	0.34	<0.01	0.92	0.54	0.95
Leptothorax sp.2	42	0.16	0.03	30.60	0.25	<0.01	0.84	0.56	0.86
Mymica carinata	39	0.21	0.04	14.10	0.09	<0.01	0.76	0.55	0.82

Myrmecina nipponica	86	0.26	0.03	26.40	0.20	<0.01	0.83	0.62	0.86
Myrmica kotokui	54	0.35	0.03	88.90	0.86	<0.01	0.99	0.57	1.00
Myrmica kurokii	35	0.23	0.02	81.50	0.79	<0.01	0.99	0.56	1.00
Pachycondyla chinensis	50	0.30	0.05	30.40	0.23	<0.01	0.83	0.57	0.85
Pachycondyla javana	252	1.91	0.06	58.90	0.55	<0.01	0.95	0.96	0.61
Paratrechina flavipes	285	2.68	0.07	57.00	0.53	<0.01	0.94	0.97	0.59
Pheidole fervida	214	1.75	0.08	44.10	0.40	<0.01	0.79	0.75	0.64
Polyrhachis lamellidens	23	0.11	0.03	9.59	0.04	<0.01	0.80	0.53	0.88
Ponera scabra	43	0.12	0.02	15.70	0.10	<0.01	0.83	0.57	0.91
Pristomyrmex pungens	181	1.09	0.06	43.30	0.39	<0.01	0.90	0.83	0.79
Stenamma owstoni	23	0.09	0.02	34.10	0.28	<0.01	0.94	0.54	1.00
Strumigenys lewisi	88	0.28	0.03	31.10	0.25	<0.01	0.83	0.63	0.86
Tetramorium tsushimae	55	0.24	0.04	35.40	0.28	<0.01	0.82	0.57	0.85
Vollenhovia emeryi	83	0.31	0.04	27.20	0.23	<0.01	0.82	0.61	0.84
